# Ethnomedicinal, phytochemical, pharmacological, and conservation studies of an endangered plant: the desert teak (*Tecomella undulata* (Sm.) Seem.)

**DOI:** 10.3389/fphar.2025.1665446

**Published:** 2025-11-17

**Authors:** Sharad Vats, Nikkee Bhandari, Showkat Ahmad Ganie, Mushtaq Ahmad Mir, Nasreena Bashir

**Affiliations:** 1 Department of Bioscience and Biotechnology, Banasthali Vidyapith, Vanasthali, Rajasthan, India; 2 Department of Clinical Biochemistry, Kashmir University, Srinagar, India; 3 Department of Clinical Laboratory Sciences, College of Applied Medical Sciences, King Khalid University, Abha, Saudi Arabia

**Keywords:** *T. undulata*, endangered plant, ethnomedicine, pharmacology, phytochemistry, conservation

## Abstract

*Tecomella undulata* (Sm.) Seem, an endangered plant, is native to India, Afghanistan, Iran, Oman, and Pakistan. Traditionally, in India, the stem bark is commonly used for the treatment of leucorrhea, pain, sexual disorders, digestive disorders, eczema, and skin infections. On the other hand, in Pakistan, both the flowers and stem bark are used as a remedy for different ailments (hepatitis, jaundice, sexual disorders, anorexia, constipation, and menstrual disorders). Phenolic metabolites and their derivatives, flavonoids, steroids, alkaloids, terpenoids, fatty acids and their derivatives, and quinones are the primary bioactive metabolites identified from this plant using different spectral and chromatographic techniques. *T. undulata* possesses hepatoprotective, antimicrobial, analgesic, antidiabetic, antioxidant, anti-obesity, acaricidal, and miticidal activities. However, these bioactivities have been partially validated scientifically. Thus, comprehensive reports exploring the mechanism of action of plant extracts/metabolites are needed to ascertain the therapeutic effect of *T. undulata*. The use of the plant in Ayurvedic formulations, as a source of timber, and in a few patents highlights their commercial importance. Preliminary toxicity studies suggest that the plant is reasonably safe; however, more in-depth data from animal models and clinical studies are needed to confirm its safety. There are a few reports on the micropropagation of this endangered plant, which can be used as a conservation strategy. With the plant being included in the Red Data Book, it becomes imperative to explore its tissue culture for the sustainable production of leading bioactive metabolites. Overall, this review compiles information on the ethnomedicinal uses, bioactive metabolites, pharmacology, commercial applications, toxicity, and micropropagation of *T. undulata* for further exploitation of the plant as a therapeutic agent.

## 1 Introduction

India is known to have a rich biodiversity of medicinal plants, which possess significant social, economic, and cultural value (Hamilton, 2004). Since ancient times, local communities have utilized botanical remedies for various health issues. Medicinal plants play a vital role in Ayurveda, Unani, Siddha, homeopathy, and naturopathy, offering numerous healing benefits. Moreover, they form a crucial component of the botanical drug industry and traditional medicine, providing income support to many in developing countries (Kumar et al., 2011). The therapeutic properties of plants are often attributed to the presence of bioactive metabolites, specifically secondary metabolites (Kumar and Janagam, 2011; Kandar, 2021). Even today, more than 60% of newly approved drugs are derived from natural sources, highlighting the relevance of secondary metabolites in both traditional and modern pharmacology (Li and Vederas, 2009).

The industrial relevance of medicinal plants has led to their depletion in the wild, which is a global issue. The current rate of extinction of plant species outnumbers the rate of natural extinction by 100–1,000 times (Wilson, 1988). The updated IUCN Red List includes 26,840 endangered species out of a total of 96,951 species (IUCN, 2021). Thus, it is essential to protect and rationally use the phyto-diversity for the sustainable development of human society (Orme et al., 2005). The enhanced extinction of endangered plants may seriously affect entire ecosystems and is a matter of concern for survival and human development. Thus, conservation of plant resources, including scarce and endangered species, is crucial for maintaining the diversity of Earth’s biological systems (Cyranoski, 2008). Plant tissue culture plays a pivotal role in agriculture, horticulture, metabolites, and conservation sectors (Pithiya et al., 2022). This technique involves the propagation of plants *in vitro* on a nutrient medium under aseptic conditions, allowing for the generation of multiple plants from a single explant (Vats et al., 2024).

*Tecomella undulata* (Sm.) Seem, commonly known as Rohida, honey tree, desert teak, Marwar teak, or white cedar, belongs to the Bignoniaceae family. This monotypic genus is native to India, Afghanistan, Iran, Oman, and Pakistan. *T. undulata* thrives in well-drained loamy to sandy loam soils with a pH range of 6.5–8.0, making it well-suited for arid environments. This species is adapted to low-rainfall areas, typically receiving between 150 and 500 mm of annual precipitation. It can endure significant temperature variations and shows remarkable tolerance to extreme cold, surviving temperatures as low as 0 °C to −2 °C in winter and reaching up to 48 °C–50 °C during summer (Singh et al., 2017). The plant has garnered interest in both classical and folk streams of the ancient medicinal system due to its therapeutic value (Ravishankar and Shukla, 2007), which is also mentioned in the ancient Samhitas of Ayurveda (Khare, 2004). Gelseminum undulatum (Sm.) Kuntze., *Bignonia undulata* (Sm.)., Tecoma undulata (Sm.) G.Don, Bignonia tropaeolum Jacquem. ex DC, and Tecoma glauca DC are synonyms of *T. undulata* (WFO, 2025). The monograph of the plant has been published in the Ayurvedic Pharmacopoeia of India (API, 2008), highlighting its therapeutic uses against helminthiasis, jaundice, skin diseases, obesity, constipation, leucorrhea, and other metabolic disorders. The tree yields good-quality timber. However, slow growth and overexploitation of this tree for medicinal and other commercial purposes have led to its classification as an endangered species (POWO, 2025).

Overall, *T. undulata* is an important medicinal plant, but there appears to be a dearth of manuscripts establishing the connection between its ethnopharmacological uses, phytochemistry, and modern pharmacological investigations. To date, no comprehensive review has been published to elucidate the limitations of studies on the plant, including its safety and toxicity, micropropagation strategies, and future perspectives. Therefore, this review aims to comprehensively summarize the ethnomedicinal importance, phytometabolites, bioactivities, toxicity, commercial importance, and *in vitro* propagation reports. The authors believe that this review is significant as it will help researchers identify research gaps and plan further strategies to establish *T. undulata* as a promising candidate for future drug discovery.

## 2 Geographical distribution and botanical description

**FIGURE 1:**
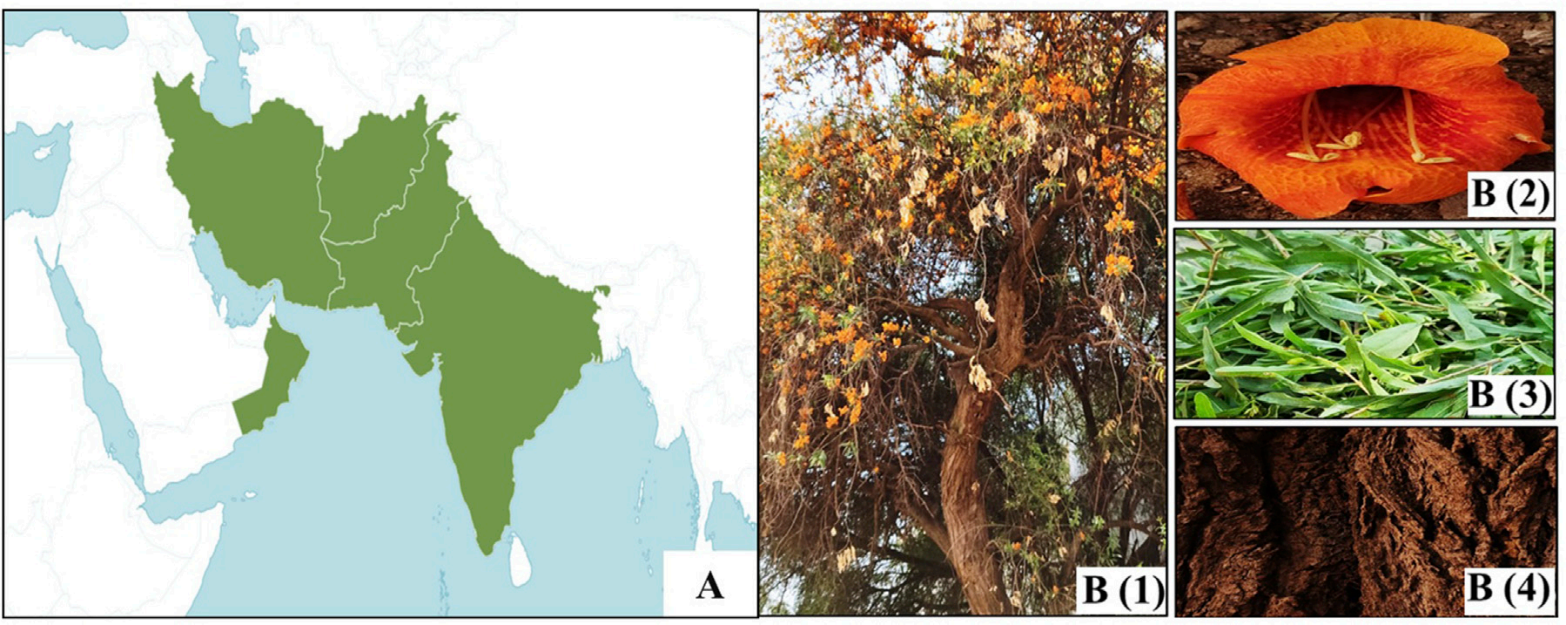
**(A)** Distribution of *T. undulata* (POWO, 2025). **(B)** Whole plant (1), flower (2), leaf (3), and bark (4) of *T. undulata*.

The tree thrives in arid regions across parts of Afghanistan, Oman, southern Pakistan (Sindh and Baluchistan), and northwestern India (Rajasthan, Gujarat, Maharashtra, Punjab, and Haryana) (Tewari, 2007; Figure 1A). The majority of Rohida is found in western Rajasthan, particularly in districts such as Ajmer, Barmer, Bikaner, Churu, Jaisalmer, Jodhpur, Nagaur, Pali, and Sikar (Meena and Kant, 2022).

*T. undulata* has a curved trunk and drooping branches. The circumference measures 52 cm–80 cm, and the height varies from 4 to 10 m. In its natural habitat, it grows up to 8 m. The roots are deep-seated, and growth is slow. Leaves are greenish, thick, and coriaceous. Defoliation occurs from November until the end of March, but complete leaf shedding does not occur as new leaves begin to appear in mid-February (Kalia et al., 2014).

The tree produces large and showy flowers on shorter lateral branches (Figure 1B). The pedicle bases measure 1 cm–2 cm in length and are terete in shape. The calyx is yellow or green, 8 mm–9 mm long, ovate, campanulate, often recurved, and may have some black spots on the outer side. The corolla is yellow–orange (5 cm–7 cm long) and veined with five equal lobes. The anterior stamens are 10 mm–30 mm long, while the posterior stamens are 2.5 mm long, and they are exerted. The filaments are glabrous. There is a yellow annular disc ovary, a style (4.5 cm long), and a bilamellated stigma (3.6 mm long) and spathulate–oblong lobes (Arya et al., 1992). The botanical classification and morphological characteristics of *T. undulata* are summarized in Table 1.

**TABLE 1:** Botanical taxonomy and morphological characteristics of *T. undulata*.

Category	Taxonomic level	Classification (POWO, 2025)
Botanical taxonomy	Kingdom	Plantae
	Phylum	Streptophyta
	Class	Equisetopsida
	Subclass	Magnoliidae
	Order	Lamiales
	Family	Bignoniaceae
	Genus	*Tecomella*
	Species	*undulata*
Morphological traits	Leaf	Petiole: present; shape: elliptic–oblong to elliptic–lanceolate or linear–oblong; margin: undulate; midrib: prominent
	Flower	Pedicel: persistent; corolla: campanulate; number: each inflorescence containing 7–11 buds; color: pale yellow or red

## 3 Ethnomedicinal uses

*T. undulata* is an important plant used in traditional medicine. The stem bark of the plant is used in the preparation of various Ayurvedic formulations. The bark is also used in the preparation of various botanical formulations (Liv-52, Amlycure, Livo Plus, Herboliv, and Livosan) for the treatment of hepatic tissue (Jain et al., 2012). Ayurvedic massage oil and fairness masks are made from this plant, combined with other plants (Jain et al., 2012). Extracts or decoctions of powdered bark in clarified butter are beneficial in treating intestinal worms, jaundice, anemia, and urinary disorders, which may be attributed to an imbalance of pitta and *kapha* (Khare, 2004).

In India, the root pulp, along with rice water, is administered orally as treatment in the Churu district and the Shekhawati region of Rajasthan (Katewa and Galav, 2005). The Garasia tribe (Rajasthan) and tribal communities in Chhattisgarh use various parts of Rohida for treating syphilis and old wounds, respectively (Meena and Yadav, 2010). In the Aravalli Hills, the Meena tribes have reported its use in treating allergic reactions (Meena and Rao, 2010). Stem bark in combination with other plants is used to heal fractures (Paul and Prajapati, 2014). The bark oil is used to treat syphilis, eczema, and skin eruptions, and the heartwood is used for the treatment of diabetes. However, no mention of the ethnomedicinal use of oil from any part of the plant or the heartwood has been documented in Pakistan.

In Pakistan, women in the Khuzdar and Kalat regions use flowers to make tea, which sterile women consume during menstruation. A paste made from fresh leaves is applied to the forehead during headaches (Tareen et al., 2010). Bark powder (100 g) is administered daily as a tonic to procumbent animals until recovery. Bark powder is also taken with hot milk by women of the Samahni Valley for abortion (Muhammad et al., 2006). Syphilis, gonorrhea, hepatitis, conjunctivitis, infection, wounds, anorexia, jaundice, liver disorders, etc., are also treated using *T. undulata* (Table 2).

**TABLE 2:** Traditional uses of *T. undulata*.

Geographical location	Plant parts used	Mode of preparation and administration	Traditional uses	Reference
India	Stem bark	NR	Mild relaxant, cardiotonic, and chloretic activities	Saggoo et al. (2014)
		Chewed for 7 days (O)	Syphilis	Pareek and Sharma (2017)
		Chewed for 7 days (O)	Birth control	
		Powder mixed with honey and sugar and taken with milk (O)	Leucorrhea	
		One cup decoction (O)	Leucorrhea	Maru and Patel (2014)
		Extract mixed with sulfur powder and applied to skin (T)	Irritation in camels	Kumar et al. (2004)
		NR	Astringent and anti-inflammatory	Kumar and Khan (2023)
		NR	Syphilis and leucorrhea, jaundice, eye disorder, cough, cold, fever, and skin disorder	Jeph and Khan (2020)
		Powder, decoction, and extract in clarified butter (O)	Jaundice, intestinal worms, swollen spleen, anemia, and urinary disorders	Khare (2004)
		Paste (T)	Wounds and conjunctivitis	Dhir and Shekhawat (2012)
		Juice administered in eyes (T)	Conjunctivitis	
		Powder (NR)	Piles, anorexia, flatulence, tumors, and intestinal worms, and as a digestive stimulant	
		NR	Fever, cough, digestive disorder, skin infection, and analgesic	Kumar and Khan (2023)
		Decoction (O)	Anorexia	Wagh and Jain (2019)
		Paste (T)	Eczema	Singh (2004)
		Powder of bark of *Sterculia urens*, *S. villosa*, *T. undulata*, and leaves of *Dalbergia volubilis* are mixed in equal proportion and soaked in water to make a paste (T)	Healing of fracture	Paul and Prajapati (2014)
		Equal proportions of bark of *T. undulata*, *Garuga pinnata*, and *Lannea coromandelica* and root of *Sterculia villosa* are crushed with a mortar and pestle to make a paste (T)		
		Powder once a day (O)	Cough and syphilis	Mewada et al. (2021)
		Decoction (O)	Sexual disease	Sen and Bhakat (2019)
		NR	Allergies and abortifacient	Meena and Rao (2010)
		Bark oil (NR). Bark oil (T)	Syphilis, eczema, and skin eruptions	Katewa (2009)
		NR	Eczema and skin eruptions	Agarwal and Rijhwani (2021)
		NR	Leucorrhea, liver disease, and diabetes	Patel and Khare (2023)
		Paste (T)	Eczema and skin eruptions	Katewa and Galav (2005)
	Stem bark of young branches	NR	Syphilis	Tripathi et al. (2000)
Pakistan	Stem bark	Powder with hot milk (O)	Abortifacient	Muhammad et al. (2006)
		Decoction/infusion (O)	Hypertension, diuretic, liver tonic, and depurative	Rahim et al. (2023)
		Decoction (O)	Anorexia	Wagh and Jain (2019)
		Powder with hot milk for few days (O)	Abortifacient	Muhammad et al. (2006)
		Decoction (T)	Leukoderma, dermatitis, abscess, wounds, and skin infection	Rehman et al. (2022)
		Decoction (O)	Constipation, stomach ache, and menstrual disorders	Tareen et al. (2010)
	Stem bark of young branches	NR	Syphilis, hepatitis, leucorrhea, and fevers	Panhwar and Abro (2007)
India	Flower	Decoction (O)	Jaundice	Wagh and Jain (2019)
		NR	Sunstroke	Soni et al. (2018)
		NR	Leucorrhea, liver disease, and diabetes	Patel and Khare (2023)
		NR	Fever, cough, digestive disorder, skin infection, and analgesic	Kumar and Khan (2023)
Pakistan	Flowers	Tea (O)	Infertility	Tareen et al. (2010)
		Decoction/infusion (O)	Hypertension, diuretic, liver tonic, and depurative	Rahim et al. (2023)
		NR	Syphilis, gonorrhea, hepatitis, tumors, conjunctivitis, blood purifier, and wound healer	Perveen et al. (2024)
		Decoction (O)	Jaundice	Wagh and Jain (2019)
		Decoction (O)	Worms, constipation, tetanus, menstrual problems, and wounds	Yaseen et al. (2018)
		Soaked in water to make extract (O)	Reduce thirst	Tareen et al. (2010)
		Tea (O)	Cure for sterility	
India	Root	Pulp with rice water (O)	Leucorrhea	Katewa and Galav (2005)
		Powder with milk (O)	Leucorrhea	Katewa (2009)
		NR	Leucorrhea	Agarwal and Rijhwani (2021)
		Powder mixed with sugar (O)	Leucorrhea	Katewa and Galav (2005)
Pakistan	Root	Pulp with rice water (O)	Leucorrhea	
		Decoction (O)	Hepatitis and eczema	Ullah et al. (2023)
India	Seeds	*T. undulata* and *Linum usitatissimum* paste applied twice everyday (T)	Abscess	Tripathi et al. (2000)
		NR	Allergies and abortifacient	Meena and Rao (2010)
Pakistan	Seeds	Crushed with leaf extract of *Pinus* (O)	Hemorrhoids	Muhammad et al. (2006)
India	Leaves	Vapors from rushed leaves	Cough	Sachdeva et al. (2023)
		NR	Syphilis and spleen disorder	Parekh and Chanda (2007)
		NR	Fever, cough, digestive disorder, skin infection, and analgesic	Kumar and Khan (2023)
Pakistan	Leaves	Decoction/infusion (O)	Hypertension, diuretic, liver tonic, and depurative	Rahim et al. (2023)
		NR	Syphilis, gonorrhea, hepatitis, tumors, conjunctivitis, blood purifier, and wound healer	Perveen et al. (2024)
		Paste (T)	Migraine	Tareen et al. (2010)
	Fresh leaves	Paste applied on head (T)	Migraine	
India	Heartwood	Soaked in water overnight and consumed (O)	Diabetes	Rohilla et al. (2016)
India	Stem	NR	Syphilis and spleen disorder	Parekh and Chanda (2007)
		NR	Syphilis and spleen disorder	
Pakistan	Shoot	Decoction (O)	Worms, constipation, menstrual problems, wounds, and tetanus	Yaseen et al. (2018)
Pakistan	Whole plant	NR	Liver and spleen disorders and tumors	Muneeb et al. (2023)

**FIGURE 2:**
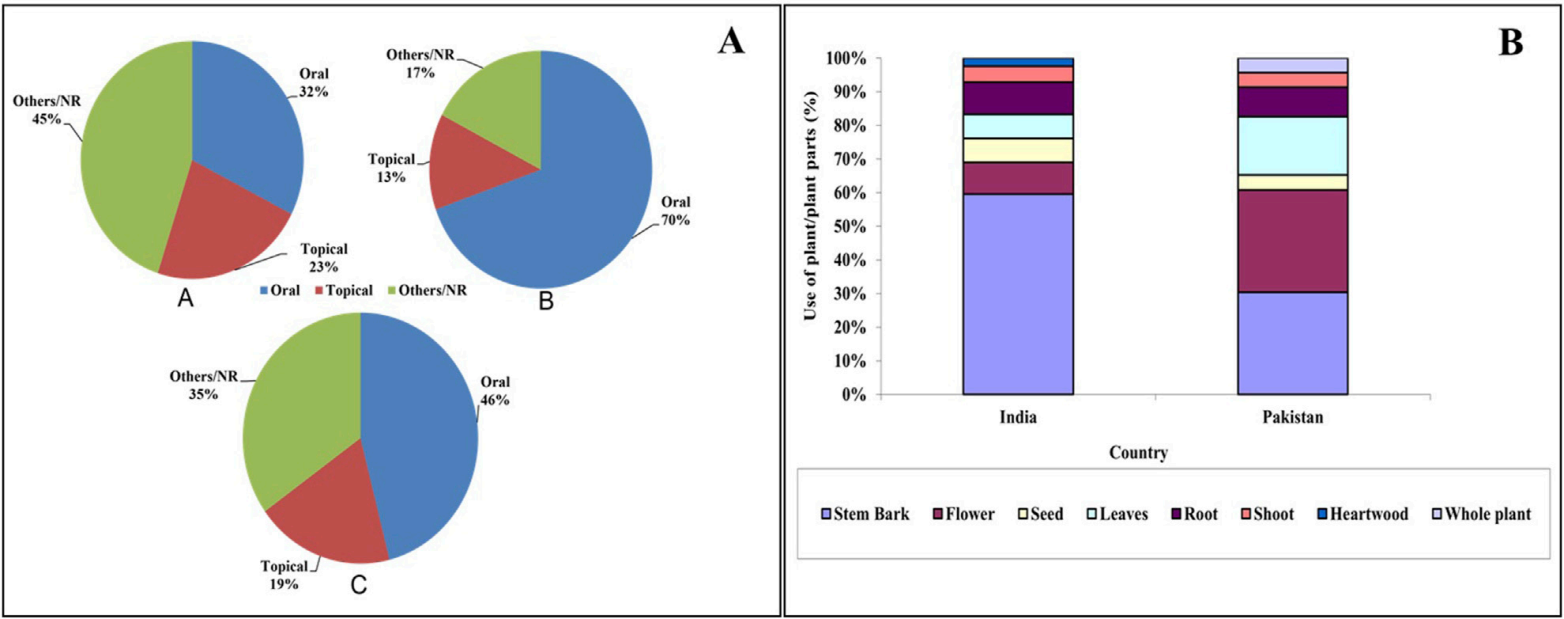
**(A)** Mode of administration of ethnomedicines from *T. undulata*. A: India; B: Pakistan; C: overall. **(B)** Use of plant parts (%) in the preparation of ethnomedicine in India and Pakistan.

Overall, the plant is most commonly used in the treatment of liver disorders, followed by leucorrhea and syphilis. Oral administration of the traditional drugs was most common in India and Pakistan (Figure 2A). Considering the plant parts, it was observed that the stem bark was used extensively in India. On the other hand, in Pakistan, the use of stem bark and flowers was almost equal (Figure 2B). Most of the data did not specify the amount of plant parts used for the traditional preparation or the duration of administration. The ethnomedicinal uses of *T. undulata* have been partially validated scientifically through pharmacological studies, which further support its potential as a promising medicinal candidate (Alvala et al., 2013; Srinivas et al., 2023; Ravi et al., 2011).

## 4 Phytochemistry

**FIGURE 3:**
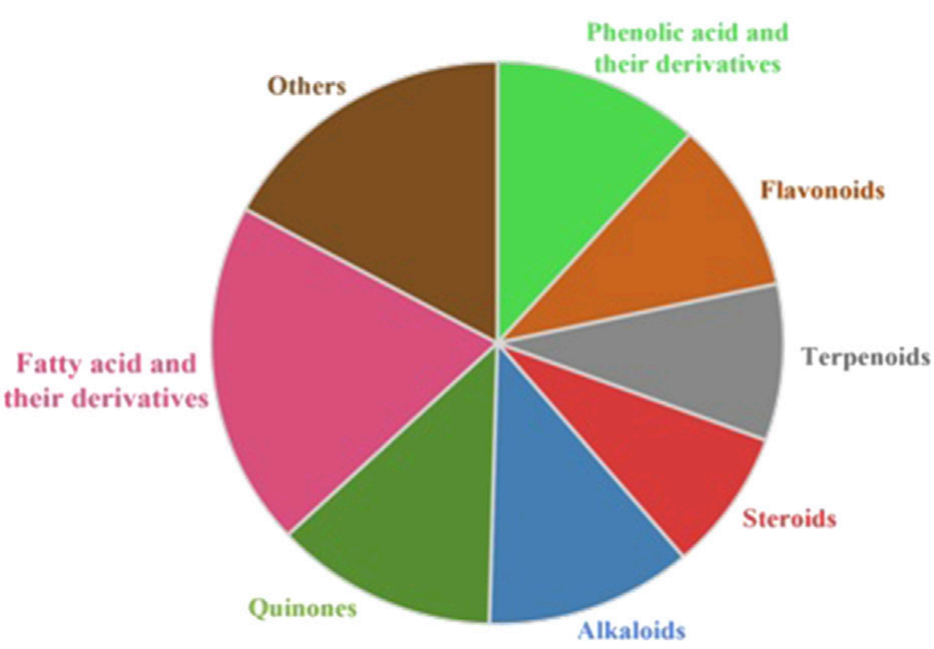
Major phytochemical groups identified in *T. undulata*.

*T. undulata* has been reported to possess several bioactive metabolites. Fatty acids and their derivatives have been comprehensively explored, followed by quinones, phenolic acids and their derivatives, and alkaloids (Figure 3). The details of the individual metabolites with their bioactivities are presented in Table 3, and the structure of the metabolites is provided in Supplementary Figure S1.

**TABLE 3:** Metabolites present in *T. undulata* (systematic names, molecular formula, and weight have been retrieved from pubchem.ncbi.nlm.nih.gov).

Class of metabolites	Systematic name (IUPAC)	Common name	Molecular formula	Molecular weight	Plant part	Mode of identification	Previously reported bioactivity	Reference
Phenolic metabolites/phenolic derivatives (6-hydroxy-2,2-dimethylbenzo[h]chromen-5-yl)-2,2-ves	3,4-Dimethoxybenzoic acid	Veratric acid	C_9_H_10_O_4_	182.17	Bark	IR, UV, and NMR	Antioxidant, anti-inflammation, anti-hypertensive, and antimicrobial	Singh et al. (1972)
						GC–MS		Bhardwaj (2018)
					Root	Co-TLC and IR		Joshi et al. (1977) Duke (2014)
	(E)-3-(4-Hydroxy-3-methoxyphenyl)prop-2-enoic acid	Ferulic acid	C_10_H_10_O_4_	194.18	Bark	Column chromatography and RP-HPLC	Analgesic, choleretic, antiviral, antiallergic, antibacterial, anticancer, hepatoprotective, antidysmenorrheic, fungicide, antimutagenic, herbicide, antioxidant, hypolipidemic, immunostimulant, insectifuge, pesticide, and uterosedative	Alvala et al. (2013) Duke (2014)
	4-Hydroxy-3-methoxybenzoic acid	Vanillic acid	C_8_H_8_O_4_	168.15	Stem bark	Column chromatography, TLC, IR, UV, and ^1^H and ^13^C NMR	Antioxidant, anti-inflammatory, and neuroprotective	Ali et al. (2017) Duke (2014)
	Benzoic acid	Benzoic acid	C_7_H_6_O_2_	122.12	Root	GC–MS	Antibacterial, antipyretic, choleretic, fungicide, flavor, insectifuge, and pesticide	Bhardwaj (2018) Duke (2014)
	Phthalic acid	1,2-Benzenedicarboxylic acid	C_8_H_6_O_4_	166.13	Leaf	GC–MS	Plasticizers	Bhardwaj (2018) Sokołowski et al. (2024)
	1-*O*-butyl 2-*O*-octyl benzene-1,2-dicarboxylate	1,2-Benzenedicarboxylic acid, butyl octyl ester	C_20_H_30_O_4_	334.4	Leaf	GC–MS	NR	Bhardwaj (2018)
	2,4-di*tert*-butylphenol	Phenol, 2,4-bis(1,1-dimethylethyl)-	C_14_H_22_O	206.32	Leaf	GC–MS	Antioxidant, antifungal, and insecticide	Bhardwaj (2018) Duke (2014)
	Octacosyl (E)-3-(4-hydroxy-3-methoxyphenyl)prop-2-enoate	Cluytylferulate	C_38_H_66_O_4_	586.9	Heartwood	TLC, IR, and ^1^H NMR	NR	Singh et al. (2008) Duke (2014)
	-	n-Eicosanyl cinnamate	C_30_H_50_O_2_	442	Stem bark	Column chromatography, TLC, IR, UV, and ^1^H and^13^C NMR	NR	Ali et al. (2017)
	4-Hydroxy-3-methoxybenzoate 7-O-β-D-galacturunofuranosyl-(2a→1b)-O-β-D-glucofuranosyl-(2b→1c)-O-β-D-arabinopyranosyl-(2c→1d)-O-β-D-[(arabinopyranosyl)8]--(2k→1L)-O-β-D-arabinopyranoside	Vanillic acid dodecaglycoside	C_70_H_107_O_55_	1827	Stem bark	Column chromatography, TLC, IR, UV, ^1^H and ^13^C NMR	Anticancer, anti-obesity, antidiabetic, antibacterial, anti-inflammatory, and antioxidant	Ali et al. (2017) Duke (2014)
	5-(6-Hydroxy-2,2-dimethylbenzo[h]chromen-5-yl)-2,2-dimethylbenzo[h]chromen-6-ol	Tectol	C_30_H_26_O_4_	450.5	Root	Co-TLC and IR	Antiplasmodial	Joshi et al. (1977)
					Heartwood	TLC and ^1^H NMR		Joshi et al. (1986) Chemfaces (2025)
	-	Octacosanyl acetoxyferulate	C_40_H_68_O_5_	628.96	Heartwood	TLC, IR, and ^1^H NMR	NR	Joshi et al. (1986)
	Octacosyl (*E*)-3-(4-hydroxy-3-methoxyphenyl)prop-2-enoate	Octacosanyl ferulate	C_38_H_66_O_4_	586.9	Heartwood	TLC and ^1^H NMR	Anti-inflammatory	Joshi et al. (1986) Duke (2014)
Flavonoid	[(2R,3S,4S,5R,6S)-6-[5,7-dihydroxy-2-(4-hydroxyphenyl)-4-oxochromen-3-yl]oxy-3,4,5-trihydroxyoxan-2-yl]methyl (E)-3-(4-hydroxyphenyl)prop-2-enoate	Tiliroside	C_30_H_26_O_13_	594.5	Flower and leaf	HPLC–ESI–MS/MS	Antioxidant, antiproliferating, free radical scavenger agent, anti-HIV, diaphoretic, and protisticide	Laghari et al. (2013); Luhata and Luhata (2017)
	2-Methyl-5,7-dihydroxychromone 7-0-β-D-glucopyranoside	-	C_16_H_18_O_9_	354.31	Bark	Column chromatography, IR, UV, and NMR	NR	Gujral et al. (1979)
	2-(3,4-Dihydroxyphenyl)-3,5,7-trihydroxychromen-4-one	Quercetin	C_15_H_10_O_7_	302.23	Bark	Column chromatography and RP-HPLC	Analgesic, antiaging, antibacterial, anticariogenic, antidiabetic, antiviral, antihypertensive, antimalarial, antimelanomic, fungicide, antioxidant, COMT-inhibitor, copper-chelator, hepatoprotective, and HIV-RT-inhibitor	Alvala et al. (2013); Duke (2014)
	-	5,6-Dimethyoxy-3′,4′-dioxymethylene-7-O-(6″-β-D-glucopyranosyl-β-D-glucopyranosyl) Flavanone	-	-	Leaf and flower	HPLC–ESI–MS/MS	NR	Laghari et al. (2013)
	-	Genistein 4′,7-O-diglucoside methylmalonylated	-	-	Leaf and flower	HPLC–ESI–MS/MS	NR	Laghari et al. (2013)
	5-Hydroxy-3-[2-hydroxy-4-[(2*S*,3*R*,4*S*,5*S*,6*R*)-3,4,5-trihydroxy-6-(hydroxymethyl)oxan-2-yl]oxyphenyl]-6-(3-methylbut-2-enyl)-7-[(2*S*,3*R*,4*S*,5*S*,6*R*)-3,4,5-trihydroxy-6-(hydroxymethyl)oxan-2-yl]oxychromen-4-one	Luteone 4′,7-O-diglucoside	C_32_H_38_O_16_	678.6	Leaf and flower	HPLC–ESI-MS/MS	NR	Laghari et al. (2013)
	-	Luteolin 3′,4′-dimethylether-7-O-β-d-glucoside	-	-	Leaf and flower	HPLC–ESI-MS/MS	NR	Laghari et al. (2013)
	2-(3,4-Dihydroxyphenyl)-5,7-dihydroxy-3-[(2S,3R,4S,5S,6R)-3,4,5-trihydroxy-6-[[(2R,3R,4R,5R,6S)-3,4,5-trihydroxy-6-methyloxan-2-yl]oxymethyl]oxan-2-yl]oxychromen-4-one	Rutin	C_27_H_30_O_16_	610.5	Bark	Column chromatography, RP-HPLC	Antiaggregant, anti-apoplectic, antibacterial, anticancer, anti-dementia, antidiabetic, antihypertensive, anti-inflammatory, antimalarial, antioxidant, antiviral, antiprotozoal, pesticide, hepatoprotective, insecticide, hypotensive, radioprotective immunomodulator, and PAF-inhibitor	Alvala et al. (2013)
					Leaf and flower	HPLC–ESI–MS/MS		Laghari et al. (2013) Duke (2014)
	5-Hydroxy-2-(4-hydroxy-3-methoxyphenyl)-6,7-dimethoxychromen-4-one	Cirsilineol	C_18_H_16_O_7_	344.3	Leaves	Column chromatography, TLC, UV, EIMS, and ^1^H NMR	Anticancer, antioxidant, anti-inflammatory, and antiviral	Azam and Ghanim (2000) Duke (2014)
	5-Hydroxy-2-(4-hydroxyphenyl)-6,7-dimethoxychromen-4-one	Cirsimaritin	C_17_H_14_O_6_	314.29	Leaves	Column chromatography, TLC, UV, EIMS, and ^1^H NMR	Antioxidative, anti-inflammatory, antiallergic, nephroprotective, antimicrobial, anti-breast cancer, and antidepressant	Azam and Ghanim (2000) Duke (2014)
	5,7-dihydroxy-3-(4-hydroxyphenyl)chromen-4-one	Genistein	C_15_H_10_O_5_	270.24	Flowers and leaves	HPLC–ESI–MS/MS	Abortifacient, anti-inflammatory, antiaggregant, antimicrobial, and antioxidant	Laghari et al. (2013) Duke (2014)
Steroid	(3*S*,8*S*,9*S*,10*R*,13*R*,14*S*,17*R*)-17-[(2*R*,5*R*)-5-ethyl-6-methylheptan-2-yl]-10,13-dimethyl-2,3,4,7,8,9,11,12,14,15,16,17-dodecahydro-1*H*-cyclopenta[a]phenanthren-3-ol	β-Sitosterol	C_29_H_50_O	414.7	Bark	Co-TLC, IR	Antimicrobial, anti-inflammatory, immunomodulatory, antioxidant, anticancer, antifertility, antidiabetic, anti-nociceptive, anticolitic, and anti-atherosclerosis	Singh et al. (1972)
					Root	Co-TLC, IR		Joshi et al. (1977)
					Heartwood	TLC, IR		Singh et al. (2008)
					Stem, root, leaf, and bark	GC–MS		Bhardwaj (2018) Duke (2014)
	Ethyl (4*R*)-4-[(3*R*,5*S*,7*R*,8*R*,9*S*,10*S*,12*S*,13*R*,14*S*,17*R*)-3,7,12-trihydroxy-10,13-dimethyl-2,3,4,5,6,7,8,9,11,12,14,15,16,17-tetradecahydro-1*H*-cyclopenta[a]phenanthren-17-yl]pentanoate	Ethyl iso-allocholate	C_26_H_44_O_5_	436.6	Bark	GC–MS	Antimicrobial	Bhardwaj (2018) Malathi et al. (2016)
	(3S,8S,9S,10R,13R,14S,17R)-17-[(E,2R,5S)-5-ethyl-6-methylhept-3-en-2-yl]-10,13-dimethyl-2,3,4,7,8,9,11,12,14,15,16,17-dodecahydro-1H-cyclopenta[a]phenanthren-3-ol	Stigmasterol	C_29_H_48_O	412.7	Heartwood	TLC, IR	Anticancer, anti-osteoarthritis, antidiabetic, antifungal immunomodulatory, antiparasitic, antibacterial, antioxidant, antiviral, neuroprotective, sedative, antihepatotoxic, anti-inflammatory, antiophidic, estrogenic artemicide, cancer-preventive, and hypocholesterolemic	Singh et al. (2008)
					Stem	GC–MS		Bhardwaj (2018) Duke (2014)
	(8*S*,9*S*,10*R*,13*R*,14*S*,17*R*)-17-[(*E*,2*R*,5*S*)-5-ethyl-6-methylhept-3-en-2-yl]-10,13-dimethyl-2,3,4,7,8,9,11,12,14,15,16,17-dodecahydro-1*H*-cyclopenta[a]phenanthren-3-ol	Stigmasta-5,22-dien-3-ol	C_29_H_48_O	412.7	Root, bark, and leaf	GC–MS	NR	Bhardwaj (2018)
	[(3*S*)-17-[(*E*)-5-ethyl-6-methylhept-3-en-2-yl]-10,13-dimethyl-2,3,4,7,8,9,11,12,14,15,16,17-dodecahydro-1*H*-cyclopenta[a]phenanthren-3-yl] acetate	Stigmasta-5,22-dien-3-ol, acetate, (3.beta.)-	C_31_H_50_O_2_	454.7	Root and stem	GC–MS	NR	Bhardwaj (2018)
	(8*S*,9*S*,10*R*,13*R*,14*S*,17*R*)-10,13-dimethyl-17-[(2*R*)-6-methylheptan-2-yl]-6,7,8,9,11,12,14,15,16,17-decahydro-1*H*-cyclopenta[a]phenanthrene	Cholesta-2,4-diene	C_27_H_44_	368.6	Stem	GC–MS	Stimulate DNA release (neutrophil extracellular traps formation)	Bhardwaj (2018) Al-Hassan et al. (2024)
	[10,13-Dimethyl-17-(6-methylheptan-2-yl)-2,3,8,9,11,12,14,15,16,17-decahydro-1*H*-cyclopenta[a]phenanthren-3-yl] benzoate	Cholesta-4,6-dien-3-ol, benzoate, (3.beta.)-	C_34_H_48_O_2_	488.7	Stem and leaf	GC–MS	NR	Bhardwaj (2018)
	[10,13-Dimethyl-17-(6-methylheptan-2-yl)-2,3,4,7,8,9,11,12,14,15,16,17-dodecahydro-1*H*-cyclopenta[a]phenanthren-3-yl] propanoate	Cholest-5-en-3-ol (3.beta.)-, propanoate	C_30_H_50_O_2_	442.7	Stem, root, and leaf	GC–MS	NR	Bhardwaj (2018)
	Stigmast-5-en-3β-ol-3-O-β-D-arabinopyranosyl-4′4'→2a)-dihydrolapachyl-2′, 3′-didecanoate (β-sitosterol arabinosyldihydrolapachyl diester	β-Sitosterol arabinosyldihydrolapachyl diester	C_69_H_109_O_9_	1,082.59	Stem bark	Column chromatography, TLC, IR, UV, ^1^H and ^13^C NMR	NR	Ali et al. (2017)
Alkaloids	(S)-(+)-2-Pyrrolidinemethanol	2-Pyrrolidinemethanol	C_5_H_11_NO	101.15	Flowers	GC–MS	NR	Laghari et al. (2014)
	5-amino-1H-pyrazole-4-carbonitrile	3-Amino-4-pyrazole carbonitrile	C_4_H_4_N_4_	108.1	Flowers	GC–MS	NR	Laghari et al. (2014)
	3-[(2S)-1-methyl-pyrrolidin-2-yl] pyridine	3-(1-Methyl-2-pyrrolidinyl)pyridine	C_10_H_14_N_2_	162.23	Flowers	GC–MS	NR	Laghari et al. (2014)
	2-Methyl-6-propylpiperidine	2-Methyl-6-propylpiperidine	C_9_H_19_N	141.25	Flowers	GC–MS	NR	Laghari et al. (2014)
	2-Piperidin-1-yl ethanol	1-Piperidineethanol	C_7_H_15_NO	129.20	Flowers	GC–MS		Laghari et al. (2014)
	1,3-Dimethyl-2-sulfanylidene imidazole-4-carbaldehyde	4-Formyl-1,3-dimethyl-1,3(2H)-dihydroimidazole-2-thione	C_6_H_8_N_2_OS	156.21	Flowers	GC–MS	NR	Laghari et al. (2014)
	5-Acetyl-1,3-diazinane-2,4,6-trione	5-Acetylpyrimidine-2,4,6(1H,3H,5H)-trione	C_6_H_6_N_2_O_4_	170.12	Flowers	GC–MS	NR	Laghari et al. (2014)
	1-(cyclohexen-1-yl)pyrrolidine	1-(1-Cyclohexen-1-yl) pyrrolidine	C_10_H_17_N	151.25	Flowers	GC–MS	NR	Laghari et al. (2014)
	1,2,3,4,4*a*,5,6,7,8,8*a*-Decahydroquinoline	Decahydroquinoline	C_9_H_17_N	139.24	Flowers	GC–MS	NR	Laghari et al. (2014)
	5,7-Dimethyl-1,3-diazatricyclo[3.3.1.1^3,7^]decan-6-one	5,7-Dimethyl-1,3-diazadamantan-6-one	C_10_H_16_N_2_O	180.25	Flowers	GC–MS	NR	Laghari et al. (2014)
	5-Methyl-2-phenyl-4*H*-pyrazol-3-one	2,4-Dihydro-5-methyl-2-phenyl-3H-pyrazol-3-one	C_10_H_10_N_2_O	174.20	Flowers	GC–MS	NR	Laghari et al. (2014)
	Docos-1-ene	1-Docosene	C_22_H_44_	308.6	Leaf	GC–MS	NR	Bhardwaj (2018)
	Hexadecyl (*E*)-3-(3,4-dihydroxyphenyl)prop-2-enoate	n-Hexadecanyl caffeate	C_25_H_40_O_4_	404.6	Stem bark	Column chromatography, TLC, IR, UV, and ^1^H NMR	NR	Ali et al. (2017)
Fatty Acid, -esters, - aldehydes, and -alcohols	Tetradecanal	Myristyl aldehyde	C_14_H_28_O	212.37	Stem	GC–MS	NR	Bhardwaj (2018)
	Dodecanal	Dodecanal	C_12_H_24_O	184.32	Root	GC–MS	Irritant, flavor	Bhardwaj (2018) Duke (2014)
	(*Z*)-Octadec-9-enal	9-Octadecenal, (Z)-	C_18_H_34_O	266.5	Leaf	GC–MS	Antibacterial	Bhardwaj (2018) Saravanakumar et al. (2015)
	Octadecanal	Octadecanal	C_18_H_36_O	268.5	Leaf	GC–MS	NR	Bhardwaj (2018)
	(*Z*)-hexadec-9-enal	Cis-9-hexadecenal	C_16_H_30_O	238.41	Stem	GC–MS	Antifungal, antimelanogenic, and anti-inflammatory	Bhardwaj (2018) Hoda et al. (2020)
	Hexadecanoic acid	Palmitic acid	C_16_H_32_O_2_	256.42	Bark	GC–MS	5-Alpha-reductase-inhibitor, anti-alopecic, anti-androgenic, anti-fibrinolytic, antioxidant, nematicide, and hemolytic	Bhardwaj (2018) Duke (2014)
	(*E*)-Hexadec-2-enoic acid	n-Hexadecanoic acid	C_16_H_30_O_2_	254.41	Stem and root	GC–MS	Antioxidant, antibacterial, anticancer, antifungal, and anti-inflammatory	Bhardwaj (2018) Aparna et al. (2012)
	Ethyl hexadecanoate	Hexadecanoic acid and ethyl ester	C_18_H_36_O_2_	284.5	Stem, root, and bark	GC–MS	Antioxidant, hemolytic, hypocholesterolemic, flavor, nematicide, and anti-androgenic	Bhardwaj (2018) Tyagi and Agarwal (2017)
	Bis(2-methylpropyl) 2,2-dihydroxypropanedioate	Di isobutyl 2,2-dihydroxy malonate	C_11_H_20_O_6_	248.27	Stem	GC–MS	NR	Bhardwaj (2018)
	Undecyl octadecanoate	Undecanyl stearate	C29H58O2	438.8	Stem bark	Column chromatography, TLC, IR, UV, ^1^H and ^13^C NMR	NR	Ali et al. (2017)
	Butyl octadecanoate	Octadecanoic acid and butyl ester	C_22_H_44_O_2_	340.6	Leaf	GC–MS	NR	Bhardwaj (2018)
	Butyl hexadecanoate	Hexadecanoic acid and butyl ester	C_20_H_40_O_2_	312.5	Leaf	GC–MS	Antioxidant, hypocholesterolemic, nematicide, and pesticide	Bhardwaj (2018) Duke (2014)
	Octadecane hydrazide	Stearic acid hydrazide	C_18_H_38_N_2_O	298.5	Bark	GC–MS	Cosmetics	Bhardwaj (2018) Duke (2014)
	Octadec-9-enoic acid	9-Octadecenoic acid (Z)-	C18H34O_2_	282.5	Stem and leaf	GC–MS	Anti-inflammatory, anti-androgenic, and anemiagenic properties	Bhardwaj (2018) Krishnamoorthy and Subramaniam (2014)
	Ethyl (*E*)-hexadec-9-enoate	Ethyl 9-hexadecenoate	C_18_H_34_O_2_	282.5	Root and bark	GC–MS	NR	Bhardwaj (2018)
	(8*E*,11*E*,14*E*)-icosa-8,11,14-trienoic acid	8,11,14-Eicosatrienoic acid, (Z,Z,Z)-	C_20_H_34_O_2_	306.5	Root	GC–MS	NR	Bhardwaj (2018)
	Ethyl icosanoate	Eicosanoic acid and ethyl ester	C_22_H_44_O_2_	340.6	Root	GC–MS	NR	Bhardwaj (2018)
	Heptacosan-1-ol	1-Heptacosanol	C_27_H_56_O	396.7	Leaf	GC–MS	Antimicrobial and antioxidant	Bhardwaj (2018) Duke (2014)
	Undecan-1-ol	1-Undecanol	C_11_H_24_O	172.31	Root	GC–MS	NR	Bhardwaj (2018)
	[(*E*)-dodec-2-enyl] 2,2,2-trifluoroacetate	Trans-2-Dodecen-1-ol, trifluoroacetate	C_14_H_23_F_3_O_2_	280.33	Root	GC–MS	NR	Bhardwaj (2018)
	Triacontan-1-ol	Triacontanol	C_30_H_62_O	438.8	Root	TLC and UV	Antidermatitic, antiherpetic, and anti-inflammatory	Joshi et al. (1977) Duke (2014)
	Docosan-1-ol	1-Docosanol	C_22_H_46_O	326.6	Leaf	GC–MS	Antiviral	Bhardwaj (2018) Katz et al. (1991)
Terpenoids	1R,3aS,5aR,5bR,7aR,9S,11aR,11bR,13aR,13bR)-9-hydroxy-5a,5b,8,8,11a-pentamethyl-1-prop-1-en-2-yl-1,2,3,4,5,6,7,7a,9,10,11,11b,12,13,13a,13b-hexadecahydrocyclopenta[a]chrysene-3a-carboxylic acid	Betulinic acid	C_30_H_48_O_3_	456.7	Bark	TLC, column chromatography, IR, UV, and HPLC	Anthelmintic, antibacterial, anticancer, antiedemic, anti-HIV, anti-inflammatory, antileukemic, antimalarial, antitumor, antiviral, and hepatoprotective	Jain et al. (2012) Duke (2014)
	(4*aS*,6*aR*,6*bS*,8*aR*,12*aR*,14*aR*,14*bS*)-4,4,6*a*,6*b*,8*a*,11,11,14*b*-octamethyl-1,2,3,4*a*,5,6,7,8,9,10,12,12*a*,14,14*a*-tetradecahydropicene	Olean-12-ene	C_30_H_50_	410.7	Leaf	GC–MS	Anti-inflammatory, antioxidant, and cytotoxic	Bhardwaj (2018) Duke (2014)
	(*E*,7*R*,11*R*)-3,7,11,15-tetramethylhexadec-2-en-1-ol	Phytol	C20H40O	296.5	Stem and leaf	GC–MS	Cancer-preventive and antitumor	Bhardwaj (2018) Duke (2014)
	1,2,4*a*,6*a*,6*b*,9,9,12*a*-octamethyl-2,3,4,5,6,6*a*,7,8,8*a*,10,11,12,13,14*b*-tetradecahydro-1*H*-picene	Urs-12-ene	C_30_H_50_	410.7	Bark	GC–MS	NR	Bhardwaj (2018)
	Dioctyl benzene-1,2-dicarboxylate	Di-n-octyl phthalate	C24H38O_4_	390.6	Stem	GC–MS	NR	Bhardwaj (2018)
	(5-Formyl-5,9-dimethyl-14-tetracyclo[11.2.1.0^1,10^.0^4,9^]hexadecanyl)methyl acetate	Kauran-18-aL, 17-(acetyloxy)-, (4.beta.)-	C_22_H_34_O_3_	346.5	Bark	GC–MS	NR	Bhardwaj (2018)
	1-*O*-heptyl 2-*O*-tridec-2-ynyl benzene-1,2-dicarboxylate	Phthalic acid, heptyl tridec-2-yn-1-yl ester	C_28_H_42_O_4_	442.6	Stem	GC–MS	Anti-inflammatory and antimicrobial	Bhardwaj (2018) Duke (2014)
	(*E*)-3,7,11,15-Tetramethylhexadec-2-en-1-ol	3,7,11,15-Tetramethyl-2-hexadecen-1-ol	C_20_H_40_O	296.5	Stem, root, bark, and leaf	GC–MS	Anti-perspirant, cosmetics, and fragrance ingredient	Bhardwaj (2018) McGinty et al. (2010)
	2,6,10-Trimethyl pentadecane	2,6,10-Trimethyl, 14-ethylene-14-pentadecane	C_18_H_38_	254.5	Stem, root, bark, and leaf	GC–MS	NR	Bhardwaj (2018)
	(*E*)-3,7,11,15-Tetramethylhexadec-2-ene	2-Hexadecene, 3,7,11,15-tetramethyl-, [R-[R*,R*-(E)]]-	C_20_H_40_	280.5	Stem and root	GC–MS	NR	Bhardwaj (2018)
Quinones	3,4-Dihydro-2,2-dimethyl-2H-naphtho[2,3-b]pyran-5,10-dione	Alpha-lapachone	C_15_H_14_O_3_	242.27	Heartwood	TLC, UV, IR, and ^1^H NMR	Anti-neoplastic and anti-trypanosoma	Singh et al. (2008) Duke (2014)
	3,4-Dihydro-2,2-dimethyl-2H-naphtho(1,2-b)pyran-5,6-dione	Beta-lapachone	C_15_H_14_O_3_	242.27	Heartwood	TLC, UV, IR, and ^1^H NMR	Anticancer, anti trypanosomic, reverse transcriptase inhibitor, and topoisomerase-I-inhibitor	Singh et al. (2008) Duke (2014)
	2,2-Dimethylbenzo[g]chromene-5,10-dione	Dehydro-α-lapachone	C_15_H_12_O_3_	240.25	Root	TLC, IR, UV, and NMR	Dehydrogenase inhibitor, succinate-dehydrogenase-inhibitor, alcohol dehydrogenase inhibitor, antivascular, antfungal, insectifuge, pesticide, and termitifuge	Joshi et al. (1977)
					Heartwood	TLC and ^1^H NMR		Joshi et al., 1986
						TLC, UV, IR, and ^1^H NMR		Singh et al. (2008) Duke (2014)
	2-Hydroxy-3-(3-methylbut-2-en-1-yl)naphthalene-1,4-dione	Lapachol	C_15_H_14_O_3_	242.27	Root	Co-TLC	Abortifacient, allergenic, analgesic, antibacterial, anticarcinomic, antiedemic, anti-flu, antimalarial, antiretroviral, antiviral, clastogenic, contraceptive, emetic, estrogenic, fungicide, immunosuppressant, insectifuge, pesticide, protisticide, schistosomicide, termiticide, and uterotrophic	Joshi et al. (1977)
					Bark	Column chromatography, Co-TLC, and IR		Singh et al. (1972)
						TLC, column chromatography, IR, UV, and HPLC		Jain et al. (2012)
					Heartwood	^13^C and ^1^H NMR		Singh et al. (2008) Duke (2014)
						TLC, IR, UV, and ^1^H NMR		
	7-Hydroxy-1-(2-methylprop-1-en-1-yl)benzo[g]benzo[5,6]cyclohepta[1,2,3-cd]benzofuran-8,13-dione	Radermachol	C_24_H_16_O_4_	368.4	Heartwood	Column chromatography, RP-HPLC, UV, IR, TLC, and EIMS	NR	Singh et al. (2008)
	2-Methylanthracene-9,10-dione	Tectoquinone	C_15_H_10_O_2_	222.24	Heartwood	TLC and ^1^H NMR	Insectifuge, pesticide, and termitifuge	Joshi et al. (1986) Duke (2014)
	23,23-Dimethyl-12-(2-methylprop-1-enyl)-13,22-dioxahexacyclo[12.12.0.0^2,11^.0^4,9^.0^15,20^.0^21,26^]hexacosa-1(14),2(11),4,6,8,15,17,19,21(26),24-decaene-3,10-dione	Tecomaquinone-I	C_30_H_24_O_4_	448.5	Heartwood	TLC,UV, IR, and ^1^H NMR	NR	Singh et al. (2008)
	2-Prop-1-en-2-ylbenzo[f][1]benzofuran-4,9-dione	2-Isopropenylnaphtho[2,3-b]furan-4,9-quinone	C_15_H_10_O_3_	238.24	Heartwood	TLC, IR, and ^1^H NMR	NR	Singh et al. (2008)
	2-*n*-tetracosanyl - 7,8 - dimethoxy - 3 - (1″,4''– dimethoxy - 7″- hydroxy-(3 → 2″)-naphthyl) naphthoquinone	Tetracosanylundulatol	C_48_H_68_O_7_	757.04	Stem bark	Column chromatography, TLC, IR, UV, and ^1^H and ^13^C NMR	NR	Ali et al. (2017)
	7,8-Dimethoxy −3 (2′)-(1′,4′-dimethoxy-7′-hydroxyl-(3→2′)- naphthyl)-naphthoquinone-7′-O-β-D-glucopyranosyl-(2a→1b)-O-β-D-glucopyranosyl-(2b→1c)-O-β-D-glucopyranosyl-(2c→1d)-O-β-D-glucopyranoside	Undulatoltetraglucoside	C_48_H_61_O_27_	1,069.98	Stem bark	Column chromatography, TLC, IR, UV, and ^1^H and ^13^C NMR	NR	Ali et al. (2017)
	2,7-Dimethoxy-3-(6′β-hydroxynonan-1′-oxy)-naphtho-1,4-quinone	Tecomella naphthoquinone A	C_21_H_28_O_6_	376.44	Stem bark	Column chromatography, TLC, IR, UV, and ^1^H and ^13^C NMR	NR	Ali et al. (2017)
	2,7-Dimethoxy-3-(12′β -hydroxypentadecan-1′-oxy)-naphtho-1,4-quinone	Tecomella naphthoquinone B	C_27_H_40_O_6_	460.60	Stem bark	Column chromatography, TLC, IR, UV, and ^1^H and^13^C NMR	NR	Ali et al. (2017)
	2-(3-Methylbut-2-enyl)naphthalene-1,4-dione	Deoxylapachol	C_15_H_14_O_2_	226.27	Heartwood	TLC and ^1^H NMR	Allergenic, pesticide, and termitifuge	Joshi et al. (1986) Duke (2014)
	(5E)-5-(2,2-dimethyl-6-oxobenzo[h]chromen-5-ylidene)-2,2-dimethylbenzo[h]chromen-6-one	Dehydrotectol	C_30_H_24_O_4_	448.5	Bark	Co-TLC and IR	NR	Singh et al. (1972)
					Root	Co-TLC, IR, and UV		Joshi et al. (1977)
						TLC and ^1^H NMR		Joshi et al. (1986)
Others	(1*S*,13*R*,15*R*,16*S*,18*R*)-9,15-dimethoxy-5,7,17-trioxa-12-azahexacyclo[10.6.2.0^1,13^.0^2,10^.0^4,8^.0^16,18^]icosa-2,4(8),9-triene	Undulatin	C_18_H_21_NO_5_	331.4	Bark	TLC, IR, UV, and ^1^H NMR	NR	Verma et al. (1986)
	[(1S,2S,4S,5S,6R,10S)-2-(hydroxymethyl)-10-[(2S,3R,4S,5S,6R)-3,4,5-trihydroxy-6-(hydroxymethyl)oxan-2-yl]oxy-3,9-dioxatricyclo[4.4.0.02,4]dec-7-en-5-yl] 3,4-dimethoxybenzoate	6-0-veratryl catalposide	C_24_H_30_O_13_	526.5	Root	Co-TLC and IR	NR	Joshi et al. (1977)
					Heartwood	IR, UV, NMR, and MS		Joshi et al. (1975)
	Pentacosa-10,12-diynoic acid	10–12-Pentacosadiynoic acid	C_25_H_42_O_2_	374.6	Stem	GC–MS	NR	Bhardwaj (2018)
	1-Methyl-2-(3-methylpentyl)cyclopropane	Cyclopropane, 1-methyl-2-(3-methylpentyl)-	C_10_H_20_	140.27	Stem and bark	GC–MS	NR	Bhardwaj (2018)
	Icosylcyclohexane	Cyclohexane, eicosyl-	C_26_H_52_	364.7	Stem, root, bark, and leaf	GC–MS	NR	Bhardwaj (2018)
	2-Methylpentadec-1-ene	1-Pentadecene, 2-methyl-	C_16_H_32_	224.42	Stem and root	GC–MS	NR	Bhardwaj (2018)
	Nonadecan-2-one	2-Nonadecanone	C_19_H_38_O	282.5	Stem	GC–MS	Anti-inflammatory and antidepressant	Bhardwaj (2018) Duke (2014)
	(*E*)-7-methylundec-3-ene	3-Undecene, 7-methyl-, (E)-	C_12_H_24_	168.32	Root	GC–MS	NR	Bhardwaj (2018)
	3-Cyclohexylpropan-1-ol	Cyclohexanepropanol-	C_9_H_18_O	142.24	Root	GC–MS	NR	Bhardwaj (2018)
	Dioctyl 3-decylbenzene-1,2-dicarboxylate	1,2-Benzenedicarboxylic acid, dioctyl ester	C_34_H_58_O_4_	530.8	Root and bark	GC–MS	NR	Bhardwaj (2018)
	8-Methyldec-1-ene	1-Decene, 8-methyl-	C_11_H_22_	154.29	Bark	GC–MS	NR	Bhardwaj (2018)
	[(*Z*)-tridec-4-enyl] acetate	Z-4-Tridecen-1-yl acetate	C_15_H_28_O_2_	240.38	Bark	GC–MS	NR	Bhardwaj (2018)
	3*a*,4,5,6,7,7*a*-Hexahydro-2-benzofuran-1,3-dione	1,3-Isobenzofurandione, hexahydro	C_8_H_10_O_3_	154.16	Bark	GC–MS	NR	Bhardwaj (2018)
	Dibutyl benzene-1,2-dicarboxylate	1,2-Benzenedicarboxylic acid, dibutyl ester	C_16_H_22_O_4_	278.34	Leaf	GC–MS	NR	Bhardwaj (2018)
	2-*O*-cycloheptyl 1-*O*-(4-methylpentyl) benzene-1,2-dicarboxylate	Phthalic acid, cycloheptylisohexyl ester	C_21_H_30_O_4_	346.5	Leaf	GC–MS	NR	Bhardwaj (2018)
	1-*O*-pentyl 2-*O*-tridec-2-ynyl benzene-1,2-dicarboxylate	Phthalic acid, pentyl tridec-2-yn-1-yl ester	C_26_H_38_O_4_	414.6	Leaf	GC–MS	Uric acid inhibitor	Bhardwaj (2018) Kundu and Sinha (2023)
	Heptadecyl 2,2,3,3,4,4,4-heptafluorobutanoate	Heptadecyl heptafluorobutyrate	C_21_H_35_F_7_O_2_	452.5	Leaf	GC–MS	NR	Bhardwaj (2018)
	Heptadecyl 2,2,2-trifluoroacetate	Heptadecyl trifluoroacetate	C_19_H_35_F_3_O_2_	352.5	Leaf	GC–MS	NR	Bhardwaj (2018)
	(*E*)-6-methylundec-2-ene	2-Undecene, 6-methyl-, (E)-	C_12_H_24_	168.32	Leaf	GC–MS	NR	Bhardwaj (2018)

### 4.1 Phenolics and flavonoids

Phenolic metabolites and flavonoids show potent antioxidant activity by scavenging ROS and free radicals through enzyme inhibition, metal chelation, and hydrogen donation. These metabolites regulate the metabolism, inflammation, and immune responses and are also utilized in the treatment of diabetes, cardiovascular disorders, and viral infections (Yao et al., 2024).

Various metabolites belonging to different groups, including phenolics (vanillic acid and its derivatives, n-eicosanyl cinnamate), were isolated from the methanolic extract of the stem bark using column chromatography followed by thin-layer chromatography. The study used triple chemical fingerprinting methods to characterize the isolated metabolites (Ali et al., 2017). Alkaloid extract, when analyzed by GC–MS, yielded several phenolic metabolites, including veratric acid, benzoic acid, and phthalic acid, from different plant parts (Bhardwaj, 2018). In another study, the root was successively extracted with petroleum ether and acetone. The former extract yielded acidic and neutral metabolites. The latter showed the presence of tectol. Joshi et al. (1986) further identified tectol and octacosanyl ferulates in the heartwood using the above methodology. On extracting the ether-soluble fraction of the stem bark with sodium carbonate, further acidification showed the presence of veratric acid using TLC and other spectral techniques (Singh et al., 1972). Different extraction techniques were employed to isolate flavonoids from the hydroethanolic extracts of *T. undulata* flowers and leaves using HPLC–ESI–MS/MS. In all the techniques, leaves had more content of individual flavonoids except 5,6-dimethyoxy-3′,4′-dioxymethylene-7-O-(6″-β-D-glucopyranosyl-β-D-glucopyranosyl) flavanone, which had less content in leaves when extracted through Soxhlet, marination, sonication, and reflux. The highest content of rutin was found to be in flowers (28.1%) and leaves (28.2%). The lowest content was that of luteolin glucoside in leaves processed through marination (Laghari et al., 2013). Among the various extraction techniques, microwave-assisted extraction (MAE) yielded the highest flavonoid content in the shortest time. The MAE technique significantly reduces energy consumption, solvent usage, and processing time, making it a sustainable and environmentally friendly methodology. It should be preferably coupled with conventional extraction to enhance the extraction yield and purity (Alvi et al., 2022). Cirsimaritin and cirsilineol were reported in the petroleum ether extract of leaves separated through column chromatography (Azam and Ghanim, 2000). The ethyl acetate fraction of the stem bark was found to contain ferulic acid (4.95%), quercetin (0.72%), and rutin (0.18%), all of which have been shown to have anti-obesity activity (Alvala et al., 2013). Quercetin was also identified in the stem bark, having antiproliferative activity (Ravi et al., 2011).

### 4.2 Steroids

The perhydrocyclopentanophenanthrene nucleus forms the basic skeleton of steroid molecules. Numerous types of steroids exist due to variations in this fundamental skeleton, and the attachment of different functional groups results in structural diversity and biological activities among steroids (Atanasov et al., 2021).

The petroleum ether extract of heartwood (3 kg) was dissolved in ethyl acetate and extracted with sodium carbonate (Bhardwaj, 2018). The sodium carbonate-insoluble fraction yielded stigmasterol (1 g) and β-sitosterol (1.5 g). Sitosterol was commonly observed in the bark (Singh et al., 1972) and root (Joshi et al., 1977) following an almost similar extraction procedure. Steroidal arabinosyl diester was characterized from the methanolic extract of the stem bark using column chromatography with petroleum ether and chloroform in equal proportion, followed by TLC for further purification (Ali et al., 2017). Stigmasterol is utilized in the synthesis of semi-synthetic and synthetic pharmaceutical compounds. It demonstrates a broad spectrum of pharmacological effects. Similarly, β-sitosterol, a common dietary phytosterol, is found to inhibit tumor metastasis by enhancing gut immunity and also contributes to blood sugar regulation, immunomodulation, reproductive protection, and fever reduction (Rani et al., 2025).

### 4.3 Fatty acids, fatty esters, fatty aldehyde, and fatty alcohols

Ethyl hexadecanoate was commonly identified in the stem, root, and bark, with the highest content found in the stem bark. The GC–MS data revealed the maximum diversity in terms of the presence of fatty acids and their esters in various plant parts that were tested (Bhardwaj, 2018). Out of the five identified fatty aldehydes, the maximum content was found to be of cis-9-hexadecenal in the stem, and the minimum content was of octadecanal in the leaves. Two fatty alcohols, viz., 1-undecanol and trans-2-dodecen-1-ol, trifluoroacetate, were reported in the roots with peak areas of 2.38% and 3.71%, respectively. Triacontanol was reported by Joshi et al. (1977) in the roots of the plant. There are some limitations to the analysis of plant metabolites using GC–MS as it can only separate volatile metabolites, which are typically of low molecular weight. Non-volatile and polar metabolites should preferably be derivatized before analysis. Moreover, for proper chemical fingerprinting, it is always suggested to identify the metabolites using multiple spectral and chromatographic techniques (Heinrich et al., 2022).

### 4.4 Alkaloids

Alkaloids are nitrogen-containing bioactive substances that are promising candidates for drug development due to their significant biological and structural activity. These metabolites possess diverse therapeutic potential and are used in the treatment of cancer, inflammation, malaria, hypertension, diabetes, etc. (Rajput et al., 2022).

The flower (20 g) was processed to obtain an alkaloid fraction (0.5 g), which was then analyzed using GC–MS. During extraction, n-hexane was used instead of chloroform, which facilitated the removal of fatty metabolites and other interfering metabolites. Derivatization of the alkaloid fraction is a standard process used to achieve better results with GC–MS as it enhances the volatility, detection, and separation efficiency by chemically altering the functional groups on the original molecule (Wang et al., 2025). However, in this study, derivatization was not included. Almost 50% of the alkaloids were present in the fraction, and they were structurally diverse, comprising aromatic, cyclic, and bicyclic compounds, which is quite rare (Laghari et al., 2014). Out of 11 alkaloids identified, the largest peak area was observed for 4-formyl-1,3-dihydro-1,3-dimethyl-2H-imidazole-2-thione (16.63%), while the smallest area was for 1-piperidineethanol (1.44%). In leaves and stems, 1-docosene (Bhardwaj, 2018) and n-hexadecanyl caffeate, respectively, have also been reported (Ali et al., 2017). However, some alkaloids also have side effects/toxicity on human health (Rajput et al., 2022); thus, the use of plant-based medicines rich in alkaloids needs to undergo meticulous safety assessments.

### 4.5 Terpenoids

The defatted stem bark powder was extracted with methanol, leading to the isolation of betulinic acid, which was further purified (98%) using column chromatography and preparative TLC (Jain et al., 2012). Betulinic acid is a lupane-type pentacyclic triterpene having several bioactivities, including antidiabetic, anticancer, diuretic, antiviral, and immunomodulatory activities (Oliveira-Costa et al., 2022). Bhardwaj (2018) reported the presence of terpenoids in the alkaloid-rich fraction of plant parts. 3,7,11,15-Tetramethyl-2-hexadecen-1-ol and 2,6,10-trimethyl,14-ethylene-14-pentadecane were found to be present in all the plant parts. This study, compared to the work of Laghari et al. (2014), clearly demonstrates the importance of selecting and processing samples appropriately to obtain a fraction rich in the metabolite of interest. Moreover, the inclusion/modification of steps that remove most of the interfering molecules becomes crucial. Both studies aimed to identify alkaloids; however, Bhardwaj et al. (2014) found the presence of very few alkaloids in the alkaloid-rich fraction.

### 4.6 Quinones

The petroleum ether extract of the heartwood (3 kg) showed the presence of seven quinones. Radermachol (70 mg; rare pigment) and 2-isopropenylnaphtho [2,3-b]furan-4,9-quinone (30 mg) were reported for the first time in the genus *Tecomella* (Singh et al., 2008). Other naphthoquinone derivatives were also reported. Naphthoquinone derivatives (Tecomella naphthoquinone A and Tecomella naphthoquinone B) were reported for the first time in the stem bark of the plant by Ali et al. (2017). Dehydrotectol was reported in the bark (Singh et al., 1972) and root (Joshi et al., 1977; Joshi et al., 1986) of the plant. Lapachol is another quinone commonly found in the root, bark, and heartwood (Table 3). This metabolite has been reported to be toxic to monkeys (Willard and Murray, 2020).

### 4.7 Other metabolites

Undulatin, an iridoid glucoside, was identified in the stem bark of the plant. The defatted powdered sample was extracted with ethanol, followed by ethyl acetate, to yield undulatin (50 mg). The metabolite was characterized using IR, UV, and ^1^H NMR spectroscopy (Verma et al., 1986). Another iridoid glucoside, 6-0-veratryl catalposide, was isolated from the ether-insoluble acetone extract of the heartwood (Joshi et al., 1975) and the root of the plant (Joshi et al., 1977). Undecanyl stearate was identified from the methanolic extract of the stem bark with a 0.034% yield. Using GC–MS, Bhardwaj (2018) identified diacetylene (stem), cyclopropane derivatives (stem and bark), alkene (stem and root), ketone (stem), primary alcohol (root), phthalate esters (root, bark, and leaves), alkene (bark), anhydride (bark), fluoroalkyl (leaves), and esters (leaves).

## 5 Bioactivities

*T. undulata* has traditionally been used by indigenous healers and herbalists to treat diseases in humans and animals. The scientific validation of traditional wisdom and experiences has often highlighted the mechanisms and modes of action of plants or their extracts and confirmed the effectiveness of bioactive products. The various pharmacological activities exhibited by different parts of the plant, along with their reported effects, are listed in Table 4.

**TABLE 4:** Pharmacological properties of *T. undulata*.

Pharmacological activity	Taxonomic validation of plant	Part used	Tested extract	Model	Control	Dose range; duration	Result/effects	Reference
Protective effects on the spleen	G: Y S: Y A: N F: Y	Stem bark	Aqueous extract	Wistar albino rats	NR	200 mg/kg–1,200 mg/kg; 30 days	Decrease in the spleen size, no significant changes in the spleen histology, and improved hematological data	Nagpal et al. (2019)
Anticancer	G: Y S: Y A: Not as per MPNS F: Y	Stem bark	Chloroform extract	Cancer cell lines	Positive control (curcumin)	10 mg/kg–100 μg/mL; 24 h–48 h	Cells became apoptotic, DNA damage, exposed phosphatidylserine residues bound to Annexin V, and uptake of 7-aminoactinomycin	Ravi et al. (2011)
	G: Y S: Y A: N F: N	Aerial part	Methanol extract	Cancer cell lines HepG2 lungs A549	Positive control (gossypol)	10 mg/kg–100 μg/mL; 72 h	CC50 (117.37) CC50 (142.01)	Riaz et al. (2022)
Antimicrobial	G: Y S: Y A: Y F: Y	Stem bark	Chloroform extract	Disc diffusion	Positive control (gentamicin, tetracycline, levofloxacin, and ceftriaxone)	25 μL/disc of 200 mg/mL extract; NR	Did not inhibit any of the test organisms	Pandya et al. (2019)
			Methanol extract				Inhibited the growth of *Bacillus cereus*, *Escherichia coli*, *Klebsiella pneumonia*, and Salmonella *typhimurium*	
			Aqueous extract				Did not inhibit any of the test organisms	
	G: Y S: Y A: N F: N		Chloroform: methanol (4:1) extract loaded on PCL/PVP nanofiber mat	Disc diffusion	NR	7.5%; 24 h	Active against *P. aeruginosa, S. aureus*, and *E. coli*	Suganya et al. (2011)
	G: Y S: Y A: N F: Y	Leaves	Hexane extract	Agar well diffusion method	Positive control (ciprofloxacin), negative control (hexane, chloroform, and methanol)	100 and 300 mg/mL; NR	Active against *Klebsiella pneumonia* and *Micrococcus luteus*	Sharma A. et al. (2013)
			Chloroform extract				Inhibited *Bacillus subtilis*, *Enterococcus faecalis*, *Escherichia coli*, *Klebsiella pneumonia*, *Micrococcus luteus*, *Proteus vulgaris*, and *Pseudomonas aeruginosa* at higher doses	
			Methanol extract				Active against all the tested organisms	
	G: Y S: Y A: N F: N		Ethyl ether and 50% ethanol (1:2), reconstituted in water	Disc diffusion	Positive control (chloramphenicol, penicillin, and mycostatin); negative control (ethyl ether and 50% ethanol)	NR	Active against *Staphylococcus aureus*, *Escherichia coli*, and *Candida albicans*	Kapoor and Bansal (2013)
	G: Y S: Y A:Y F: Y		Methanolic extract	Well diffusion	NR	NR	Active against *Staphylococcus epidermidis* and *Bacillus subtilis*	Parekh et al. (2005)
	G: Y S: Y A: Not as per MPNS F: Y		Ethanolic extract	Dilution method	Positive control (ampicillin, gentamicin, ceftazidime, and erythromycin)	Different concentrations; 24 h	MIC and MBC against *Acinetobacter baumannii* (0.62 and 1.25 mg/mL, respectively)	Valizadeh et al. (2020)
Acaricidal (*Sarcoptes scabiei*)	G: Y S: Y A: Not as per MPNS F: Y	Branches	Methanol extract	Food poisoning method	Positive control (ivermectin) Negative control (methanol)	10%, 20%, and 30% of 50% stock solution; 72 h	45%, 65%, and 80% mortality of mites, respectively	Khan et al. (2013)
			Methanol extract	Topical method		10% and 20% of 50% stock solution; 5 weeks	68%, 69%, 72%, 68%, and 66% mortality of mites infecting buffalo, camel, dog, goat, and human skin, respectively, at 20% dose after the fifth week	
Hepatoprotective	G: Y S: Y A: N F:Y	Stem bark	NR	Albino Wistar rats	NR	200 and 400 mg/kg, once daily for 30 days	Decreased level of ALT, ALP, AST, GGT, and bilirubin	Zehri et al. (2020)
	G: Y S: Y A: N F: Y		Methanolic extract	Albino Wistar rats	Positive control (silymarin)	200 mg/kg; 48 h	SGOT, SGPT, ALP, TBL, and cholesterol levels decreased and total proteins increased	Rana et al. (2008)
	G: Y S: Y A: N F: Y		50% ethanolic extract	Albino rats	Positive control (N-acetylcysteine)	200 mg/kg; 15 days	Absence of necrotic tissues and mild infiltration of lymphocytes	Fatima et al. (2022)
	G: Y S: Y A: N F: Y		50% ethanolic extract	Albino rats	Positive control (N-acetylcysteine)	200 mg/kg; 15 days	Lowered AST, ALT, and ALP levels	Fatima et al. (2023)
	G: Y S: Y A: Not as per MPNS F: Y		Ethanolic extract	Wistar albino rats	Positive control (silymarin)	1,000 mg/kg; 7 days	Reduced serum AST, ALT, GGT, ALP, total bilirubin, and liver MDA levels. Improved liver glutathione. Reduced liver necrosis	Khatri et al. (2009)
	G: Y S: Y A: N F: Y		Various extracts, fractions, and isolated metabolites	HepG2 cells	Positive control (silymarin)	10 µg/mL–200 µg/mL; 24 h	Reduced ASTand ALT	Jain et al. (2012)
			Various extracts, fractions, and isolated metabolites	Wistar rats	Positive control (silymarin)	Methanolic extract (TSB-7) 200 and 400 mg/kg and betulinic acid (MS-2) 50 and 100 mg/kg; 7 days	Reduced LPO, AST, and ALT. Enhanced SOD, CAT, GSH, and AA. Lowered vacuolation, centrilobular necrosis, and nuclear condensation in liver	Jain et al. (2012)
	G: Y S: Y A: Y F: Y		Various reactions of ethanolic extract	Wistar rats	Positive control (silymarin)	150 mg/kg; 4 days	Reduced AST, ALP, ALT, and bilirubin. Increased total protein	Patel et al. (2011)
	G: Y S: Y A: Y F: Y		Methanolic extract	C57Bl/6 mice	Positive control (saroglitazar; glucose tolerance and insulin sensitivity)	80 mg/kg; 12 weeks	Reduced body weight, insulin resistance, ALT, AST, TG, TC, endoplasmic reticulum stress, inflammation, and oxidative stress. Improved antioxidant capacity. Reduced fat accumulation and lobular inflammation in hepatocytes	Srinivas et al. (2023)
	G: Y S: Y A: N F: Y	Leaves	Methanolic extract	Wistar albino rats	Positive control (silymarin)	100 mg/kg and 200 mg/kg; 15 days	Decreased AST, ALT, ALP, GGT, total bilirubin, and LPO. Increased levels of SOD, CAT, GSH, and GPx. Normal hepatic cords and absence of necrosis and vacuoles	Singh and Gupta (2011)
	G: Y S: Y A: Not as per MPNS F: Y	NR	Ethanolic extract	Wistar albino rats	Positive control (silymarin)	100 and 400 mg/kg; 14 days	Decreased levels of ALT, AST, and ALP. Enhanced GSH and SOD levels	Saxena et al. (2021)
Anti-ulcer	G: Y S: Y A: Not as per MPNS F: Y	Leaves	Ethanolic extract	Albino Wistar rats	Positive control (omeprazole)	250 mg/kg and 500 mg/kg; NR	Reduced ulcer index and ulcerated area	Arsalan et al. (2023)
Laxative	G: Y S: Y A: Not as per MPNS F: Y	Leaves	Ethanolic extract	Albino Wistar rats	Positive control (lactulose)	250 mg/kg and 500 mg/kg; NR	Increased feed intake, reduced water intake, and increased number and weight of fecal pellets	Arsalan et al. (2023)
Antidiabetic/antihyperglycemic	G: Y S: Y A: N F: N	Leaves	Ethanolic extract	Albino rats	NR	250 mg/kg and 500 mg/kg; 28 days	Reduced levels of glucose, total cholesterol, LDL, triglyceride, and VLDL; normal cellular and nuclear morphology of pancreatic islets; and liver and kidney biochemical parameters returned to normal	Lal et al. (2017a)
	G: Y S: Y A: Not as per MPNS F: Y	Heartwood	Petroleum ether, chloroform, acetone, and hydroalcoholic extract	*In vitro* assays	Positive control (acarbose)	20, 40, 60, 80, and 100 μg/mL; 10 min	Alpha-amylase and alpha-glucosidase inhibitor	Rohilla et al. (2016)
	G: Y S: Y A: Not as per MPNS F: Y	Stem bark	50% ethanolic extract	Albino Wistar rats	Positive control (glibenclamide)	250 and 500 mg/kg; 21 days	Decreased level of glucose, cholesterol, triglycerides, and LDL; protected pancreatic β-cells	Das et al. (2015)
	G: Y S: Y A: Not as per MPNS F: Y	Leaves	Ethanolic extract reconstituted in 2% Tween 80	Albino Wistar rats	Positive control (metformin)	200 and 500 mg/kg; 30 days	Glucose, Hb1Ac, and malondialdehyde levels decreased and GSH levels increased	Kumar et al. (2012)
Antiplasmodial	G: Y S: Y A: Y F: Y	Leaves	Methanolic extract	*Plasmodium falciparum*	Positive control (artemisinin)	IC 50: 15 µg/mL; 48 h	Cytotoxic	Sachdeva et al. (2023)
Anti-obesity	G: Y S: Y A: Not as per MPNS F: Y	Stem bark	Ethyl acetate extract	3T3-L1mouse fibroblasts	Dexamethasone	10 µg/mL–200 µg/mL; 24 h	Inhibition of adipocyte differentiation and decreased triglyceride levels. Reduced levels of SIRT1, PPARγ, C/EBPα, E2F1, leptin, FAS, LPL mRNA, and proteins	Alvala et al. (2013)
		Stem bark	Ethyl acetate extract	Swiss albino mice	Positive control (orlistat)	30 mg/kg/day; 1 month	Lowered cholesterol, triglycerides, LDL, LDL/cholesterol, and VLDL. Increased HDL and HDL/cholesterol	
Antiandrogenic	G: Y S: Y A: N F: Y	Leaves	Petroleum extract	Albino rats	NR	50 mg/kg–200 mg/kg; 60 days	Decreased weight of testes, epididymides, seminal vesicles, and ventral prostate. Lowered sperm motility, sperm density, testosterone, and LH. Decreased levels of protein, sialic acid, glycogen, and cholesterol in testis. Enhanced intertubular space between seminiferous tubules	Soni and Mali (2016)
Analgesic/anti-inflammatory	G: Y S: Y A: Not as per MPNS F: Y	Whole plant	Methanolic extract	Albino mice/	Positive control (aspirin)	300, 500, and 100 mg/kg; 210 min	Lowered pain (mouse tail immersion method)	Ahmad et al. (1994)
	G: Y S: Y A: Not as per MPNS F: Y	Leaves	Ethanolic extract	Albino Wistar rats	Positive control (indomethacin)	250 mg/kg and 500 mg/kg; 48 h	Reduced paw edema	Arsalan et al. (2023)

G, genus; S, specie; A, author: F, family; Y, yes; N, no; MPNS, Medicinal Plant Names Services.

### 5.1 Hepatoprotective activity

*T. undulata* is reported to have hepatoprotective activity against isoniazid-induced liver damage. The stem bark extract significantly lowered the elevated levels of AST (aspartate aminotransferase), ALT (alanine transaminase), ALP (alkaline phosphatase), GGT (gamma-glutamyl transferase), and bilirubin (Zehri et al., 2020). These enzymes are released due to the membrane damage of liver cells. Thus, the plant has a membrane-stabilizing effect. In a damaged state, the liver cannot properly release bilirubin through the bile. This may lead to its leakage into the blood. High levels of bilirubin may lead to jaundice (Bajaj et al., 2022). However, there is no clarity regarding the determination of the dose and the authentication of the plant samples. Additionally, the inclusion of histopathological studies and other markers of liver damage could have made the investigation more comprehensive. The methanolic extract of the plant reduced the levels of liver enzymes, including cholesterol, in the experimental model compared to those in the CCl_4_ group. However, the amount of protein and albumin increased. It is reported that damage to the ER leads to a loss of P540, resulting in lower protein synthesis. Moreover, CCl_4_ inhibits the synthesis of bile from cholesterol, resulting in its accumulation. Histological sections revealed that the cellular architecture improved following the administration of the plant extract (Rana et al., 2008).

In related studies, the ameliorative effect of *T. undulata* stem bark on acetaminophen-induced toxicity in rats was observed. Acetaminophen forms N-acetyl P benzoquinone (NAPQI), which is toxic. Overproduction of NAPQI generates free radicals, which damage mitochondrial DNA and increase membrane permeability, thereby adversely affecting hepatocytes. The bark extract improved liver histology, which was characterized by a reduced presence of inflammatory cells (Fatima et al., 2022), and lowered hepatic enzyme levels (Fatima et al., 2023). The leaves of the plant showed protective effects against alcohol-induced hepatotoxicity. The occurrence of liver marker enzymes decreased in the serum, and the liver GSH (glutathione), GPx (glutathione peroxidase), and SOD (superoxide dismutase) levels increased. Moreover, lipid peroxidation was also reduced. Fatima et al. did not justify the need to present the related data (histopathological and liver marker enzymes) in separate articles, which is questionable. Similar effects of the leaf extract were observed in rats with paracetamol-induced liver damage (Singh and Gupta, 2011). The extract may possess antioxidants that minimize lipid peroxidation of the membrane, and thus, the presence of marker enzymes is reduced in the serum. Alcohol decreases the production of antioxidant enzymes due to the adverse effects of free radicals or due to the production of acetaldehyde as a result of alcohol oxidation (Das et al., 2005). Jain et al. (2012) evaluated the hepatoprotective activity of the plant’s stem bark on HepG2 cells and rats and concluded that this may be due to the presence of betulinic acid (triterpenoid). TSB-2 fraction showed the highest degree of cytotoxicity, which may be due to the presence of lapachol (Almeid, 2009). TSB-7 fraction (containing betulinic acid) showed lower cytotoxicity and was assessed in an animal model. Betulinic acid is reported to be cytotoxic against several animal cell lines (Eichenmüller et al., 2009); however, HepG2 cells were less adversely affected, which may be due to the expression of survivin and Bcl2 (survival factors). The positive effect of the plant extract on liver marker enzymes is indicative of reduced liver damage and a membrane-stabilizing effect.

Non-alcoholic fatty liver disease (NAFLD) is a primary concern globally, which is caused by poor eating habits, a sedentary lifestyle, and obesity. It mainly leads to non-alcoholic steatohepatitis (NASH). Mice were fed with a Western diet sugar water (WDSW), leading to nonalcoholic steatohepatitis. In a preclinical study, the stem bark showed a positive effect on liver marker enzymes, total cholesterol levels, triglyceride levels, and insulin resistance (Srinivas et al., 2023). The mouse model mimics the effects of diet and the incidence and progression of disease in humans. The dose of the experiment was determined according to guidelines established by the US Food and Drug Administration. A pharmacopoeia evaluation of the plant material was conducted to assess its pharmaceutical quality. Overall, important factors such as the dose, mode of administration, timing of the intervention, extent of exposure, and endpoint assessments were meticulously selected to evaluate the effect of *Tecomella* in treating NASH. Oxidative stress, inflammation, and ER stress were found to be reduced, which was nearly equivalent to that of saroglitazar. Downregulation of ER stress markers [C/EBP homologous protein (CHOP), 78-kDa glucose-regulated protein (Grp78), and unfolded protein response (UPR)] was observed. The reduction in inflammation was primarily due to a decrease in pro-inflammatory markers, specifically tumor necrosis factor α (TNF-α) and interleukin-1β (IL-1β). The expression of c-Jun N-terminal kinase (JNK) and extracellular signal-regulated protein kinase (ERK1/2), which are markers of inflammation and steatosis, was observed (Urano et al., 2000). Thus, a reduction in oxidative and ER stress could have lowered the levels of cholesterol and lipids in the liver.

### 5.2 Analgesic activity

*T. undulata* exhibits notable pain-relieving properties. In a study, the whole plant was processed to obtain an extract using absolute methanol. Significant analgesic activity was observed, as assessed by the hot-water tail immersion test in mice. However, the results were not dose-dependent. The extract was not able to exert significant anti-inflammatory activity on paw edema induced by carrageenan (Ahamad et al., 1994). Carrageenan, as an irritant substance, is used to cause edema. It induces the secretion of cytokinins under the influence of bradykinin (Vats et al., 2024). The variation in results may be due to the presence and concentration of bioactive metabolite/s and pharmacokinetic variations. Since the methanolic extract was administered to animal models, it becomes imperative to include a negative control, which was not mentioned in the study.

Arsalan et al. (2023) highlighted the anti-inflammatory potential of *T. undulata* leaves. The study revealed that the plant extract was effective in both the initial and later phases of edema (Akinloye et al., 2020; Zahra et al., 2020). This may be due to the presence of phenolic metabolites in the ethanolic extract, which might have worked synergistically and antagonistically with anti- and pro-inflammatory markers. The study was carried out on formalin- and carrageenan-induced paw edema in rats. However, the study is too preliminary, and further molecular and biochemical studies are needed to establish the efficacy of the plant. However, the paw edema test is a very preliminary study and does not conclusively establish the bioactivity of the plant extract.

### 5.3 Anticancer/antimutagenic activity

Ravi et al. (2011) studied the anti-proliferative activity of the plant bark against cancer cell lines with a promising IC_50_ value (30 µg/mL) in K562 cells. The cell line exhibited characteristic features of apoptotic cells, including membrane blebs, cell shrinkage, and DNA damage. Moreover, phosphatidylserine (PS) residues were bound to Annexin V, which enhanced the uptake of 7-aminoactinomycin (7-AA). In normal cells, PS is present on the inner surface of the membrane; however, during early apoptosis, it becomes exposed on the cell surface. Annexin V is a phospholipid-binding protein with high affinity to PS (Robinson et al., 2020). On the other hand, uptake of 7-AA signifies a late apoptotic event (Wang T. et al., 2022). The chloroform extract showed the presence of a metabolite with an identical retention time to quercetin. Isolation, characterization, quantification, and evaluation of the antitumor potential of these metabolites may result in the identification of the lead target molecule.

Riaz et al. (2022) reported a bioassay-guided study of *T. undulata*, which showed significant cytotoxic, antimutagenic, and anticancer potential. The hexane extract had the greatest effect on the locomotion of *Caenorhabditis elegans*, while the methanolic extract had the least. *Salmonella typhimurium* strains TA98 and TA100 were modified for frame-shift and base-pair substitutions, respectively. The growth of these strains is inhibited in the absence of histidine in the culture medium. Plant extracts are tested in the presence of a mutagen to evaluate their antimutagenic potential, which is calculated according to the number of revertant colonies. The methanolic extract of *Tecomella* showed the highest antimutagenic potential, which may be due to the presence of flavonoids and other phenolic metabolites in the extract. Thus, the plant showed potential to prevent or inhibit the carcinogenic effect of mutagens (De Silva and Alcorn, 2019).

An MTT assay was performed to assess the cellular toxicity. The methanolic extract showed good activity against HepG2 tumor cell lines (68.17%). A significant difference in the inhibition of HepG2 cell lines was observed when comparing the data of the chloroform (Ravi et al., 2011) and dichloromethane extracts (Riaz et al., 2022) of the plant. These two solvents differ slightly in their polarity index, and the difference in their activity may be due to the time of collection and geographical location, which affect the concentrations of plant metabolites (Heinrich et al., 2022). The resazurin assay (simple, rapid, and sensitive) was performed on various cancer cell lines. The methanolic extract was found to be significantly effective on liver HepG2 and lung A549 cell lines, with CC_50_ values of 117.37 and 142.01 µg/mL, respectively. Resazurin, also known as Alamar blue, is an indicator dye used to measure cell viability. The extract was found to possess a decent selectivity index when its cytotoxicity on cancer cells was compared with that on normal cells. Such an extract or metabolite is considered suitable for further anticancer studies. The metabolites present in the extract also showed potential against cancer-related proteins, as revealed through docking studies.

### 5.4 Antidiabetic activity

The plant was reported to have a mild blood glucose-lowering effect in an acute study. The effect was quite significant in a chronic study wherein the ethanolic leaf extract was administered for 30 days. Moreover, glucose tolerance improved in streptozotocin-treated rats (Kumar et al., 2012). This may be due to the increased secretion of insulin from pancreatic cells or the peripheral utilization of glucose. In the long term, diabetes glycosylation of proteins, including hemoglobin, is observed. A high level of glycosylated hemoglobin (HbA1c) is a marker for poor glycemic control and is associated with diabetes-related disorders (Lau and Aw, 2020). The plant extract helped bring the enhanced levels of HbA1c to nearly normal levels. Insulin activates glycogen synthase to form more glycogen (Norton et al., 2022). Hepatic glycogen was found to increase after the treatment with the plant extract. This may be attributed to the reactivation of glycogen synthase in test animals. Lipid peroxidation was observed to be reduced, as evidenced by the lower levels of malondialdehyde in diabetic rats treated with *T. undulata*. Additionally, GSH levels increased in streptozotocin-induced diabetic rats. The data projects the antioxidant potential of the plant. It is known that oxidative stress occurs in diabetes. Glycosylation of proteins can lead to the generation of reactive oxygen species (ROS) (Gupta et al., 1997). In individuals with diabetes, glucose is channeled toward a pathway that requires NADPH. GSH reductase forms reduced glutathione, involving NADPH. Thus, diabetes leads to GSH depletion and enhances oxidative stress (Sha et al., 2021). However, the study authors did not highlight dose determination and toxicity analyses, which are essential in animal studies using plant extracts.

In a study conducted by Das et al. (2015), the hydroethanolic extract of the heartwood of *T. undulata* lowered blood glucose levels and serum triglycerides, total cholesterol, and low-density lipoproteins and increased high-density lipoproteins. Increased free fatty acids in diabetes are associated with decreased glucose tolerance and impaired β-cell function (Wismayer et al., 2023). Bioactives present in the plant extract may have partially reversed the damage caused to the pancreas by streptozotocin (Papuc et al., 2021), as evidenced by the histopathological study of the pancreas. A similar effect of the hydroethanolic extract was reported in streptozotocin-induced diabetic rats using the leaves (Lal et al., 2017a) and roots (Lal et al., 2017b) of the plant. *Tecomella* extract induced the rearrangement of peripheral tissue and the normalcy of islets. The biochemical studies also supported the plant’s antidiabetic efficacy. However, the mechanism of action and the identification of lead antidiabetic metabolites still need to be explored.

### 5.5 Antioxidant activity

Free radicals are byproducts of cellular metabolism. However, if their production exceeds the cell’s neutralization capacity, it leads to oxidative stress (Masenga et al., 2023). Thus, external supplementation with antioxidants is recommended, and the use of plant-based products is a matter of personal choice (Vats, 2016; Baroni et al., 2021). Bhardwaj et al. (2014) analyzed the methanolic extract of different plant parts of *T. undulata* for its antioxidant activity. The total phenolic content was found to be the highest in the stem (12.70 GAE/g DW). Meanwhile, the maximum flavonoid content was observed in leaves (71.87 mg QE/g DW). The best ferric reducing antioxidant power was observed in leaves (96.66 mM/L/g). The best antioxidant potential with respect to the ABTS assay was shown by the leaves. The results of the antioxidant assays are in accordance with the occurrence of the highest total flavonoid content in leaves. These *in vitro* antioxidant assays only define the chemical profile of a preparation and require further evaluation through pharmacological experiments to validate its antioxidant efficacy.

### 5.6 Antimicrobial activity

The methanolic extract of leaves showed better activity than the aqueous extract against *Staphylococcus epidermidis* and *Bacillus subtilis* (Parekh et al., 2005). This clearly reveals the importance of solvent selection in extracting active metabolites against a specific microbe (Nortjie et al., 2022). In another study, Sharma A. et al. (2013) reported that, among the different solvents tested, the methanolic extract exhibited the best antimicrobial potential. The least MIC (0.01 mg/mL) was observed against *Klebsiella pneumoniae*, and the highest MIC (4 mg/mL) was observed against *B. subtilis*. The antimicrobial potential of the methanolic extract of the stem bark was found to be better than that of the chloroform and aqueous extracts, as reported by Pandya et al. (2019). It is essential to note that the former study did not demonstrate any positive effect against *Salmonella typhi*; however, the latter study was found to be effective against the test organism of typhoid. The difference in the results may be due to the solubility of different bioactive metabolites in both solvents. Additionally, different plant parts may vary in terms of the type and concentration of metabolites. Moreover, the better diffusion of the methanolic extract in the microbial medium may be another reason for the differential activity (Parekh et al., 2005). Suganya et al. (2011) reported the antibacterial potential of PCL/PVP (polycaprolactone/polyvinylpyrrolidone) mats loaded with *T. undulata* extract. The medicated fibers remained stable even after high electrical voltage. The study highlighted the use of mats as both a drug carrier and a wound dressing material loaded with antimicrobials.

### 5.7 Miticidal/acaricidal/antiplasmodial activity

Khan et al. (2013) observed the maximum acaricidal activity of the methanolic extract in goats and camels, followed by humans. However, the results were better in the *in vitro* assay, wherein the plant extract (30%) showed 80% mortality. The miticidal activity may be due to the presence of lapachol, which interferes with cellular respiration and the generation of free radicals (Rahman et al., 2022). However, the actual molecular mechanism involved in the acaricidal activity needs to be further explored. Antiplasmodial activity of 17 plants was studied by Sachdeva et al. (2023). It was observed that *T. undulata* was the most effective with IC_50_ values of 15 and 15.3 µg/mL against *Plasmodium falciparum* 3D7 and *P. falciparum* INDO, respectively. Some metabolites, such as quercetin, rutin, ursolic acid, lapachol, and betulinic acid (Table 4), may be responsible for the antimalarial activity of the plant extract.

### 5.8 Anti-obesity activity

Obesity is prevalent in most parts of the world. Obesity is associated with the incidence of other disorders, viz., diabetes, cardiac disorders, and cancer (Chen et al., 2020). It has been reported that during weight loss, there is mainly a reduction in the volume of adipocytes rather than a reduction in their number (Spalding et al., 2008). The expansion of adipose tissue through hyperplasia is quite challenging to reverse as adipocytes are resistant to apoptosis. Thus, anti-obesity drugs essentially should target hyperplasia. Alvala et al. (2013) sequentially extracted the bark of *T. undulata* and further fractionated the ethyl acetate fraction. Fraction 1 (F1) significantly inhibited the division and accumulation of triglycerides in adipocytes (3T3-L1 mouse fibroblast cells). This was achieved through the activation of Sirtuin 1 (SIRT1) mRNA and proteins. In addition, downregulation of peroxisome proliferator-activated receptor gamma (PPARγ) and CCAAT/enhancer-binding protein alpha (C/EBPα) was observed. A reduction in the mRNA expression of E2F1, leptin, FAS, and LPL was also observed. On the other hand, levels of adiponectin increased. SIRT1 is known to regulate transcription factors, which affect the expression of PPARγ, C/EBPα, and leptin, among others, which in turn regulate fat metabolism. PPARγ and C/EBPα stimulate adipocyte-specific genes and regulate adipocyte differentiation. LPL expression is a marker of lipid accumulation in adipocytes. FAS enzyme catalyzes the synthesis of long-chain fatty acids, and it is upregulated during adipogenesis (Ortega et al., 2010). E2Fs regulate adipogenesis through changes in the expression of the nuclear receptor PPARγ (Chen et al., 2020). Leptin is a hormone that plays a significant role in energy balance (Tucker et al., 2024). Adiponectin is a hormone derived from fat that has been shown to affect obesity negatively (Maeda et al., 2020). Thus, agents that regulate SIRT1 activity can be essential candidates for treating obesity and its related disorders. An *in vivo* study supported the data obtained in cell lines. A significant decrease in cholesterol, triglycerides, LDL, LDL/cholesterol, and VLDL was noticed. Additionally, an increase in HDL and HDL/cholesterol was observed in mice treated with the plant extract compared to obese mice fed a high-fat diet. The F1 was found to be rich in ferulic acid (4.95%), and the metabolite has been reported to possess anti-obesity properties (Wang O. et al., 2022). However, the study did not specify how the dose administered to mice was determined.

### 5.9 Other activities

A stomach ulcer was induced in rats using ethanol, and the ulcer index was found to be 7, with an ulcerated area of 1.10 cm^2^–0.3 cm^2^ (Arsalan et al., 2023). *T. undulata* extract significantly reduced ulcer index (2) and ulcerated area (0.3 cm^2^–0.1 cm^2^). Ethanol-induced gastric ulcers cause a decline in bicarbonate secretion and a reduction in the mucus present in the gastric wall (Ibrahim et al., 2022). Ethanol induces the production of free radicals, such as the superoxide anion and hydroxyl radical, and enhances lipid peroxidation, leading to the impairment of the stomach mucosa (Ibrahim et al., 2022). Thus, the plant extract may have reduced oxidative stress, leading to decreased capillary injury, vascular permeability, and the production of inflammatory markers (Akmal et al., 2023). Furthermore, constipation was induced in rats using loperamide (Arsalan et al., 2023), which reduces peristaltic movement in the intestine, including water secretion (Parkar et al., 2024). The plant extract increased the weight and number of fecal matter, suggesting improved colon movement (Katsirma et al., 2021). This may be due to the presence of glycosides, which possess laxative activity, and their presence was also reported in the study.

The plant was projected as an antifertility agent by Soni and Mali (2016). Administration of *T. undulata* extract resulted in a 70% reduction in the weight of male reproductive organs, accompanied by a decrease in fertility rate. The sialic acid content, which facilitates the seamless movement of sperm, was found to be reduced. This may have affected the acrosomal membrane and the fertilization ability of sperm (Aslan Çetin et al., 2022). Reduced levels of luteinizing hormone and testosterone were observed. LH induces the production of testosterone. Testosterone plays a crucial role in spermatogenesis (Oduwole et al., 2021). The analysis of the phytochemicals present in the extract and the determination of the dose were not mentioned by the authors.

The ameliorative effect of *T. undulata* on CdCl_2_ (cadmium chloride)-induced splenomegaly was investigated by Nagpal et al. (2019). In rats treated with the plant leaf extract, the size of the spleen was comparable to that of the control group. The complete bold count suggested a positive effect of the plant’s extract. The hemoglobin, red blood cell, platelet, and packed cell volume data were found to be almost equal to those of the control group and better than those of the CdCl_2_-treated group. The histological studies revealed that the spleen of rats administered with CdCl_2_ showed necrosis, hyperplasia, swollen and dead cells in the pulp, and sinus congestion. These features were improved in the group treated with *T. undulata*. In the above-studied parameters, a lower dose of the plant’s extract did not prove to be effective; however, a higher dose (>600 mg/kg/day) showed promising results.

Botanical extracts, in general, are complex, and seasonal/geographical differences significantly affect the concentrations of individual metabolites, thereby affecting their efficacy. These changes are also due to the use of agrochemicals during cultivation and other variables affecting plant growth (Heinrich et al., 2022). However, none of the above-mentioned studies have provided these details.

## 6 Commercial uses

A constant surge in the demand for medicinal and aromatic plants (MAPs) has been observed globally over the past decade. Recent data suggest that the market value of MAPs was estimated to be $201 billion in 2023 and is expected to increase to $375.6 billion by 2032, with a significant compound annual growth rate (CAGR) of 7.22%. China contributed the most (22.98%) to the total worldwide exports, followed by India (10.54%). This clearly demonstrates the commercial relevance and growing interest in natural products among the general public (Zamani et al., 2025).

The bark of Rohida is extensively used in the preparation of various Ayurvedic formulations, including Rohitaka Ghrita, Rohitaka Loha, Rohitaka Rishta, and Rohitaka Dyachoorna (Jain et al., 2012). *Rohitaka Rishta* is effective in treating the liver, spleen, stomach, and skin disorders (Ullah et al., 2010). In addition, the wood of desert teak is commercially very important. It is a useful building material mainly due to its strength and durability. Thus, it is commonly used in the manufacture of furniture, such as cabinets, doors, and window frames (Kalia et al., 2014). One of the metabolites, vanillic acid, which was identified in the plant, is an oxidized form of vanillin and is used as a flavoring agent in the food industry. It is also used in the production of vanillin and the synthesis of different pharmaceutical agents. The production rate of vanillic acid has increased significantly due to the gradual increase in its demand (Kaur et al., 2022), with an estimated average growth rate of more than 1.5% (2012–2016). Moreover, the plant is utilized as an essential component in botanical formulations, which are patented and exhibit various therapeutic properties, including immune-boosting, antioxidant, anti-aging, anticancer, and antiviral effects (Table 5).

**TABLE 5:** Patents related to *T. Undulata* and their claimed properties (Google patents and https://www.wipo.int/patentscope/en/).

Application	Plant part used	Property	Publication number
Botanical drug	Stem bark	Anticancer	IN1869/MUM/2012
Botanical drug	Bark	Immunity booster	US20070122496
Botanical drug	Stem bark	Effective against corona and other viral infections	IN202011034071
Botanical drug	Stem bark	Anti-aging and antioxidant	IN42/KOL/2014

## 7 Toxicity

Cellular toxicity of the ethyl acetate fraction of *T. undulata* stem bark was assessed on 3T3-L1 mouse fibroblasts. The extract was found to be non-toxic even at a concentration of 200 µg/mL (Alvala et al., 2013). The petroleum ether extract (250 µg/mL) of the stem bark showed less than 50% viability of HepG2 cells (Jain et al., 2012); however, the methanolic extract and its fractions were found to be more viable (65%). The methanolic extract of the leaves was non-toxic to HEK293 mammalian cell lines at a concentration of 200 µg/mL (Sachdeva et al., 2023). The ethanolic extract (50%) of the plant did not cause toxicity, even at a dose of 2,000 mg/kg body weight in Wistar albino rats (Das et al., 2015). The methanolic extract and one of its metabolites, MS-2, did not exhibit toxicity at doses of 5,000 mg/kg and 1,000 mg/kg body weight, respectively (Jain et al., 2012). The aqueous extract was found to be safe up to a dose of 2,000 mg/kg body weight; however, it was toxic at doses of 4,000 and 8,000 mg/kg body weight (Nagpal et al., 2019). The methanolic extract of the bark was found to be safe up to 2,000 mg/kg body weight in terms of acute toxicity (Rana et al., 2008). Saxena et al. (2021) reported that the ethanolic extract of the bark did not exhibit toxicity up to a dose of 4,000 mg/kg body weight. One of the identified metabolites in the plant, lapachol, possesses anti-vitamin K activity. Long-term administration of lapachol (0.0625 g/kg/day–0.25 g/kg/day) in monkeys caused anemia (Willard and Murray, 2020). It is reported to induce reproductive toxicity affecting the seminal vesicle in male Wistar rats (De Cássia da Silveira e Sá and de Oliveira Guerra, 2007). Although generally safe at low concentrations, benzoic acid exhibits dose-dependent toxicities, including dermatological irritation, hypersensitivity reactions, gastrointestinal disturbances, and metabolic acidosis. At higher exposures, it may cause glycine depletion, neurological impairment, and bilirubin displacement, leading to neonatal encephalopathy (Issa and Mohammed, 2025). β-Sitosterol is generally considered safe in healthy individuals, but it may cause mild gastrointestinal effects or reduced absorption of fat-soluble vitamins at high doses (Paniagua-Pérez et al., 2005).

Even if preliminary studies on toxicity reveal that the plant is less toxic, further comprehensive studies involving animals and clinical trials are needed to ascertain the safety of *T. undulata* as a medicinal agent in healthcare. Additionally, it is worth noting that a metabolite may exhibit different activity/toxicity when administered individually or in combination with other metabolites (extract).

## 8 Micropropagation

Endangered medicinal plants typically exhibit slow growth, narrow distribution, low fruiting, poor seed development, and challenging germination. Their survival is further threatened by overharvesting and ecological changes, which hinder both slow natural regeneration and artificial reproduction. Therefore, *ex vivo* propagation is vital. Micropropagation is an important technique as it enables the rapid and large-scale production of genetically uniform, disease-free plantlets, supporting both the conservation and sustainable use of endangered medicinal species (Zheng et al., 2023).

*T. undulata* is commercially very important due to its medicinal and timber value. However, the sluggish growth and excessive cutting of this tree for commercial purposes have made it endangered. Cross-pollination has resulted in significant variability (Rathore et al., 1991) in the plant; therefore, clonal propagation of selected germplasm is desirable for conservation and for its valuable timber and medicinal properties. An overview of tissue culture studies on *T. undulata* is presented in Table 6.

**TABLE 6:** An overview of tissue culture studies on *T. undulata*.

Explant source	Callus/shooting medium; no. of shoots; shoot length	Rooting medium	Acclimatization; survival rate	Reference
Nodal shoot segments	MS + BAP (2 mg/mL) + IAA (0.05 mg/mL for bud break; 8–10; 2.18 cm further subculture MS + IAA (0.01 mg/L) + BAP (1 mg/L); 5.5; 2 cm	Shoots treated with IBA (1.5 mg/mL) and transferred to 1/2 MS liquid medium	Drained soil: vermiculite (4:1); 46%	Rathore et al. (1991)
Cotyledonary node	MS + BAP (11.09 µM)+IAA (0.57 µM); 28.3; 2.5 cm	Shoots treated with NAA (26.85 µM), IAA (28.54 µM), and IBA (24.60 µM) and transferred to ½ MS medium	Vermiculite: sand (1:1); NR	Aslam et al. (2006)
	*Agrobacterium tumefaciens* GV2260 + MS + BAP (11.09 µM) + IAA (0.57 µM); 25–30; 3–5 cm	Shoots treated with NAA (26.85 µM), IAA (28.54 µM), and IBA (24.60 µM) and transferred to ½ MS medium	Vermiculite: sand (1:1); NR	Aslam et al. (2009)
	MS + TDZ (0.7 µM) followed by subculture in MS without hormone; 43; 7.4 cm	*Ex vitro*: shoots dipped in IBA (200 µM) for 30 min, transferred in plastic cups containing soilrite, and eventually established in natural soil with 80% survival rate	Soilrite	Varshney and Anis, (2012)
Nodal segments	SNP (40 g/L); 1.82; 3.74 cm	NR	NR	Aghdaei. (2012)
	TDZ (0.1 mg/L); 2; 3.33 cm	NR	NR	
	MS + 10.0 μM BAP for bud proliferation; Schenk and Hilderbrandt medium + 5 μM BAP + 5 μM Kinetin + 50 mg/L ascorbic acid + 25 mg/L citric acid + 25 mg/L arginine; NR	Schenk and Hilderbrandt medium + 10 µM IBA + 50 mg/L Ascorbic Acid	Sand: farm yard manure (3:1) + ½ Schenk and Hilderbrandt medium; NR	Chhajer and Kalia, (2017)
	MS + NAA (0.54 μM) and BAP (8.8 μM) for bud break; MS + MS + BAP (4.4 μM); 1–2; 1.8; 20 mm	B_5_ +NAA (537.06 μM) + IBA (492.1 μM) + Ascorbic acid (567.8 μM)	Sand: compost (3:1)	Tyagi and Tomar (2013)
Callus-mediated micropropagation				
Seedling	MS + kinetin (0.1 mg/L) +NAA (1 mg/L) for callus induction; MS + IAA (0.1 mg/L) +BAP (2.5 mg/L); 20–22	Pretreatment with NAA (5 mg/L) + IBA (mg/L)+ IAA (mg/L) and transferred to ½ MS medium	Sand: vermiculite (1:1)	Nandwani et al. (1996)
Nodal	MS + kinetin (0.1 mg/L) +NAA (1 mg/L) for callus induction; MS + IAA (0.1 mg/L) +BAP (2.5 mg/L); 6–8	Pretreatment with NAA (5 mg/L) + IBA (mg/L) + IAA (mg/L) and transferred to ½ MS medium	Sand: vermiculite (1:1); 35%	Nandwani et al. (1996)

### 8.1 Shooting

Explants collected in August and September showed the best response and the highest number of shoot inductions. The maximum number of shoots was observed in MS + BAP (5 mg/mL) + IAA (0.05 mg/mL); however, the shoots were very short in length. This shows that higher concentrations of BAP inhibited the adequate differentiation of shoots (Rathore et al., 1991). It was observed that the response was better at 31 °C, possibly due to the physiological nature of the plant, which propagates optimally in a semi-arid environment. Kinetin-induced leafy shoots were not found to be better for further micropropagation steps. Furthermore, the sub-culturing of the shoots was performed on a medium with lower concentrations of auxins to inhibit callusing (Vats and Kamal, 2013; 2014; Vats, 2018). Aghdaei et al. (2012) investigated the impact of silver nanoparticles on plant culture. Nanoparticles, when used alone, showed a mild response in terms of the induction of the number of shoots (<2). In combination with hormones (BAP + IAA), the response was even poorer. When nanoparticles were used in combination with thidiazuron, the response was found to be slightly better with two shoots per explant. Thus, silver nanoparticles had no significant effect on shoot induction. This may be due to seasonal variations in terms of explant collection, region of collection, and the hormones used (Cheng et al., 2024). Aslam et al. (2006) observed a positive effect of IAA and BAP on shoot induction from cotyledonary nodes. Better shoot induction was observed when IAA and BAP were used in combination, with a superior percentage of explant response. A higher concentration of cytokinin than auxin generally promotes shoot growth (Wu et al., 2022). Chhajer and Kalia (2017) used Schenk and Hilderbrandt medium supplemented with BAP, KN, and ascorbic acid for multiple shootings. Antioxidants have been used in the medium to avoid the production of phenolic metabolites in the culture and leaching in the medium (Vats, 2018).

### 8.2 Rooting

Rooting from shoots was induced using a liquid medium supplemented with IBA for 2 days, and then the plants were transferred to half-strength MS medium (Rathore et al., 1991). The survival rate after acclimatization was evaluated to be 46%, which is comparatively lower. Aslam et al. (2006) and Aslam et al. (2009) reported a stepwise process for efficient rooting, which involved transferring *in vitro* shoots to a liquid medium containing different concentrations of NAA, IAA, and IBA, either alone or in combination, for 36 h, followed by half-strength MS medium without hormones. Many workers suggest that the two-step method reduces the number of days needed for root initiation and yields a better average number of shoots (Rathore et al., 1991; Bhansali, 1993). *In vitro-*generated shoots were cultured on Schenk and Hildebrandt (SH) medium supplemented with IBA and ascorbic acid for rooting. According to Tyagi and Tomar (2013), the most suitable medium for *in vitro* rooting was ½ B5 medium, possibly due to its lower content of ammonium nitrate and potassium nitrate than that of the MS medium. The addition of ascorbic acid improved the rooting because it acts as an antioxidant, which minimizes phenolic production involved in retarding the growth of cultures.

### 8.3 Callus

Indirect micropropagation was carried out using seedlings and nodal explants from the tree. Callus obtained from explants of the tree was hard, compact, and green; however, it was fragile and light brown when seedlings were used as explants (Nandwani et al., 1996). NAA, in combination with kinetin, had no effect on shoot induction in callus generated from both explants; however, IAA and BAP proved to be effective in this regard. Better shooting was observed in the seedling-derived callus. The two-step method was followed for root induction. Instead of supplementing hormones in agar containing MS medium, pretreatment was carried out in a hormone-containing liquid medium, and thereafter, the cells were transferred to ½ MS medium. Auxins (NAA, IBA, and IAA) were used either alone or in combination, and the best response was observed when all three hormones were used in combination at a concentration of 5 ppm. As mentioned earlier, Rathore et al. (1991) also used a two-step method; however, the response was poor. Variation in the result may be due to the difference in genotype (Holmes et al., 2021).

## 9 Conclusion and future perspectives

**FIGURE 4:**
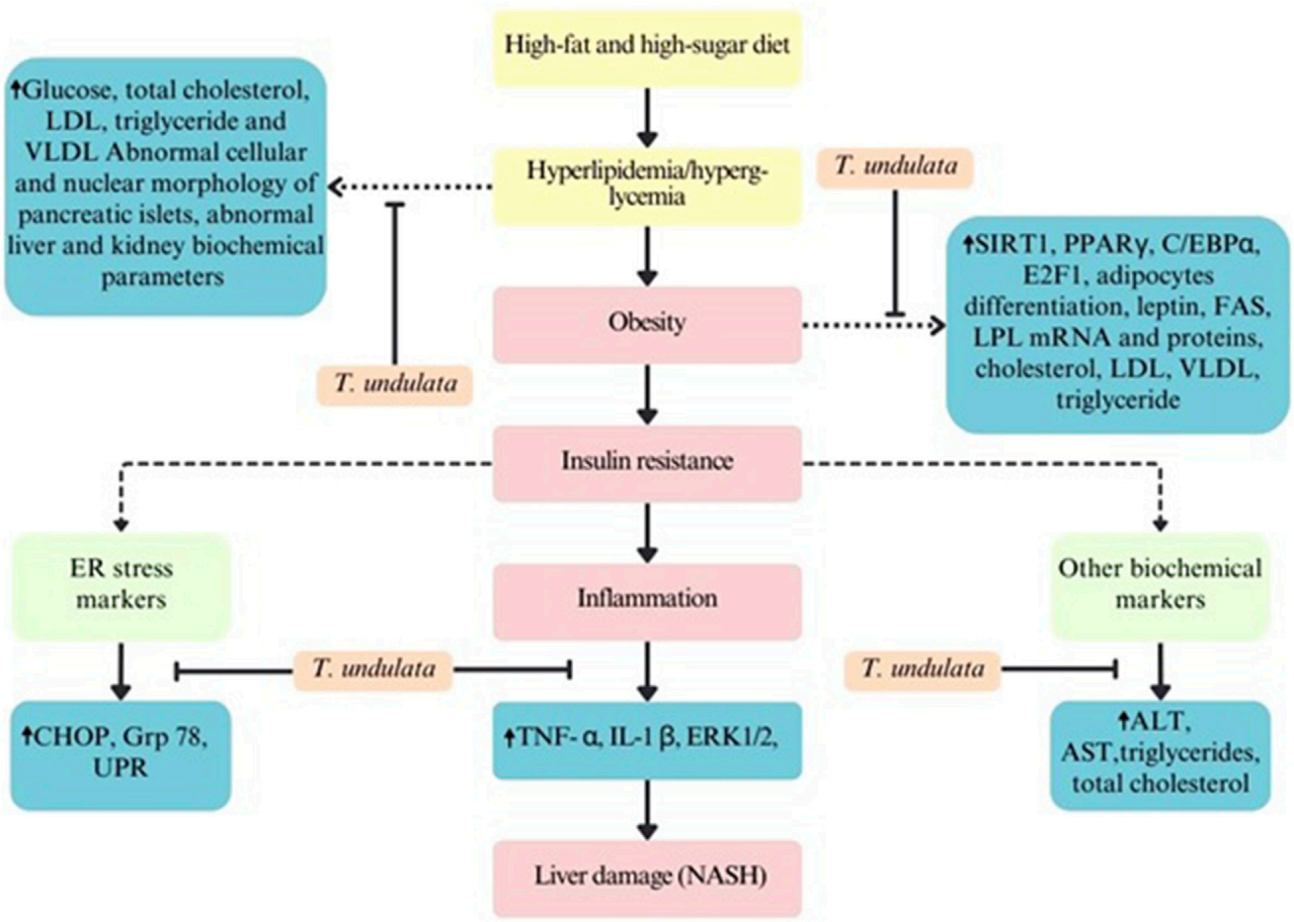
Different pharmacological activities of *T. undulata* with probable mechanisms of action.

The review summarizes the data on the botany, ethnopharmacology, phytochemicals, pharmacology, toxicity, and micropropagation of *T. undulata*. Traditionally, the plant is used to treat leucorrhea, sexual disorders, digestive disorders, liver disorders, and skin infections by the people of India and Pakistan. These activities are mainly attributed to the presence of various metabolites such as phenolic metabolites and their derivatives, flavonoids, steroids, alkaloids, terpenoids, fatty acids and their derivatives, and quinones. Certain bioactivities (hepatoprotective, antimicrobial, analgesic, antidiabetic, antioxidant, anti-obesity, acaricidal, and miticidal) have been partially validated scientifically, and a probable mode of action is provided in Figure 4. Since the plant is endangered, the review also focuses on the *in vitro* propagation techniques.

However, the following points require attention. The pharmacological activities explored in the plant are primarily focused on the stem bark, followed by the leaves. There is a dearth of reports on the bioactivities of the flowers, which have comprehensive traditional value in Pakistan. Some metabolites have been identified in *T. undulata*, but these studies may represent only a limited spectrum of the total metabolic profile. Phytochemical research primarily focuses on fatty acids, quinones, and phenolic acids, while studies on flavonoids, steroids, and terpenoids are relatively scarce. To substantiate the ethnomedicinal properties effectively, further animal and human clinical trials are required to determine the appropriate dose for treating various ailments. Bioassay-guided isolation of bioactive metabolites from different plant parts, pharmacokinetic investigations, computer-aided drug design, and elucidation of a potential mode of action are essential for exploring the therapeutic potential of key drug leads. The toxicity studies suggest that the plant is relatively safe; however, comprehensive chronic, sub-chronic, reproductive, and genotoxicity studies remain scarce. Investigating the pharmacodynamics and metabolic mechanisms will support the safe clinical use and development of more effective plant-based therapeutics.

The bioactive metabolites from the callus culture of *T. undulata* have not been identified yet, and elicitation strategies to enhance the production of secondary metabolites also remain unexplored. This will provide a comparatively sustainable approach to producing valuable metabolites.

In addition, meticulous analyses of the research articles included in the present review suggest that many studies conducted on the plant extract did not follow the guidelines of ConPhyMP (consensus-based reporting guidelines for the phytochemical characterization of medicinal plant extracts). Furthermore, studies should focus on validating the traditional uses of the plant through appropriate models (animal and cell cultures), determining the dose and selecting the most effective one, exploring the mechanism of action, and investigating various delivery systems, preferably based on nanotechnology, to ensure bioavailability and target specificity. For an unambiguous representation of data about the plant name, synonyms, identification, and distribution of plants, MPNS (The Medicinal Plant Names Services) must be referred to. These guidelines will help create an appropriate work plan, and the preclinical experimental data thus obtained can be effectively used/translated for safe and realistic clinical trials.

In summary, *T. undulata* has important ethnomedicinal and pharmacological activities. There are significant challenges related to the therapeutic application of this plant. Future studies should integrate proper taxonomic validation of the plant, mechanistic pharmacological studies, multi-omics platforms, and advanced chromatographic and spectral techniques for metabolite identification and characterization. This approach will add scientific value to the obtained data, which can further be used for clinical trials and global applications. The authors believe that this review will generate considerable interest within the scientific community and serve as a valuable reference for the future development and application of *T. undulata*.
