# Supplementary material for: Ethnomedicinal, phytochemical, pharmacological, and conservation studies of an endangered plant: the desert teak (Tecomella undulata (Sm.) Seem.)
Source: Front Pharmacol. 2025 Nov 17;16:1665446. doi: 10.3389/fphar.2025.1665446 (PMC12665970; doi:10.3389/fphar.2025.1665446)
Supplement: Supplementary file 1 [file Supplementaryfile1.doc]

**Phenolic acids/ Phenolic derivatives**

| **Benzoic acid derivatives** | | **Ferulic acid** | **n-Eicosanyl cinnamate** | **Tectol** |
| --- | --- | --- | --- | --- |
| 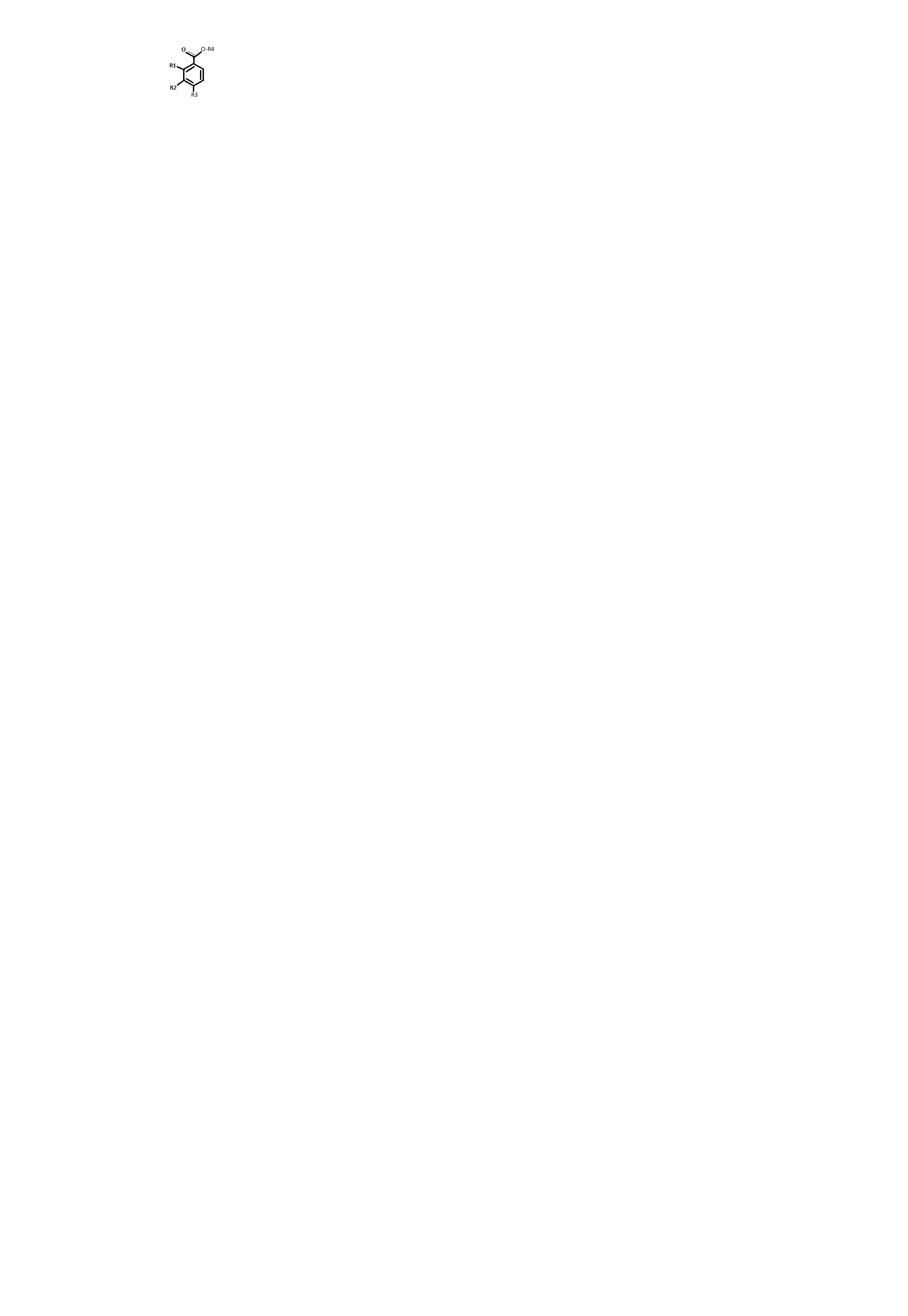 | **Veratric acid**: R2,R3:OCH_3_, R1,R4:H  **Vanillic acid**: R2:OCH_3_, R3:OH, R1,R4:H  **Phthalic acid**: R1:COOH, R 2,R3, R4:H  **1,2-Benzenedicarboxylic acid, butyl octyl ester**: R1:COO-butyl, R4:octyl | **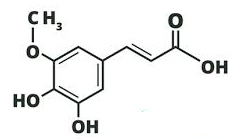** | 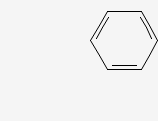 | 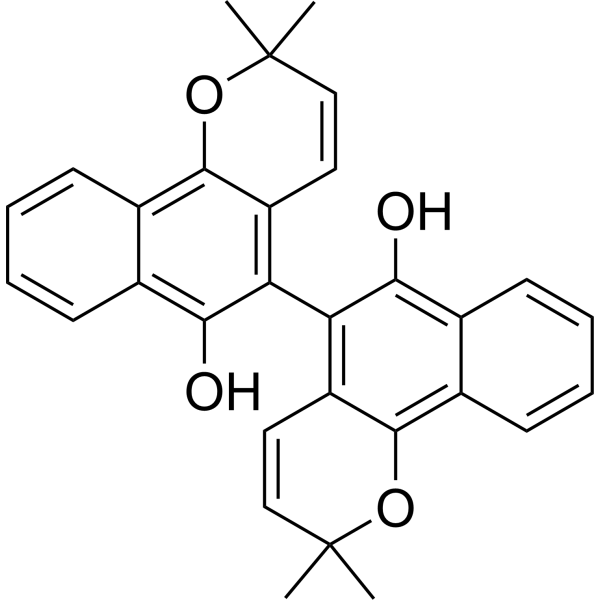 |

| Flavonoids | | | | | |
| --- | --- | --- | --- | --- | --- |
| 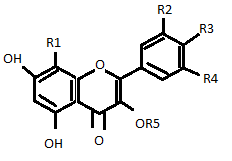Flavonol | **Quercetin**:  R1,R4:H, R2,R3:OH  Rutin:  Quercetin R5: α-L-rhamnopyranosyl-  (1→6)-β-D-glucopyranose  **Tiliroside**:  R1,R2,R4:H,  R3:OH  R5: β-D-(6''-p-coumaroyl) glucopyranoside | 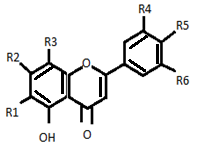Flavone | **Cirsimaritin**  R1,R2:OCH3:  R3,R4,R6:H,  R5:OH  **Luteolin**:  R1,R3,R6:H,  R2,R4,R5:OH  **Cirsilineol**:  R1,R2,R5:OCH3,  R3,R4:H,R6:OH | 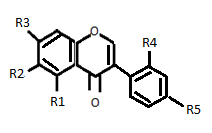Isoflavone | **Genistein**:  R1,R3,R5:OH; R2,R4:H  **Genistein 4’,7-O-diglucoside methyl malonylated:**  R1:OH,R2,R4:H,;  R3:O-Glucoside,  R5:O-methymalonylated glucoside  **Luteone 4’,7-O-diglucoside**  R1,R4:OH,R2:(CH3)2C=CH-CH2  R3,R5:O-glucoside |

| **Sterols** | | | | | |
| --- | --- | --- | --- | --- | --- |
| **β-sitosterol**  **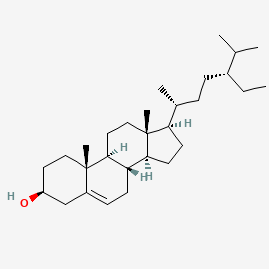** | **Ethyl iso allocholate**  **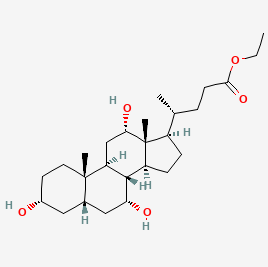** | **Stigmasterol**  **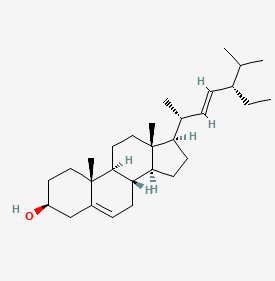** | **Stigma-5,22-dien-3-ol,acetate,(3.beta)**  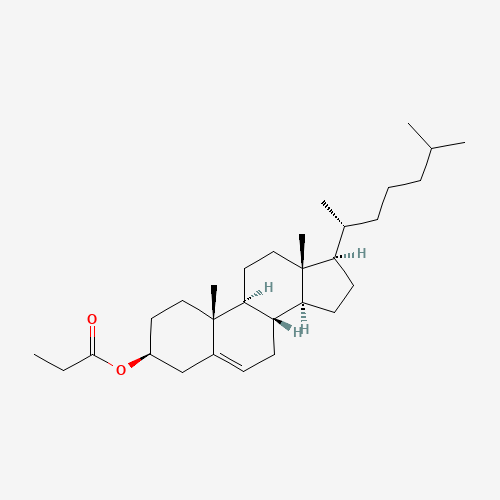 | **Cholesta-2,4-diene**  **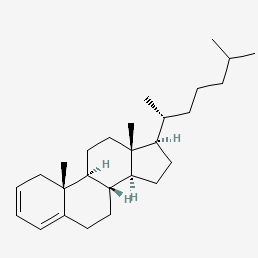** | **Cholesta-5-ene-3β-ol, propanoate**  **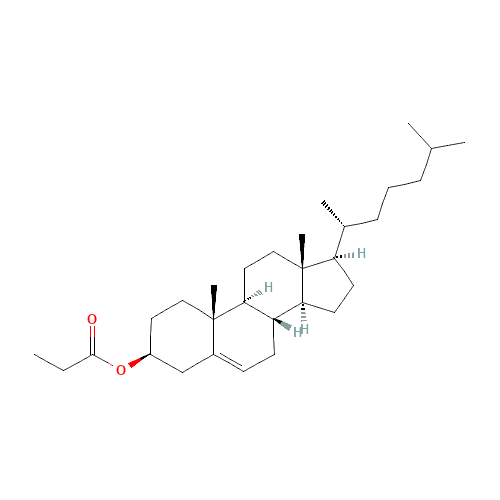** |

| **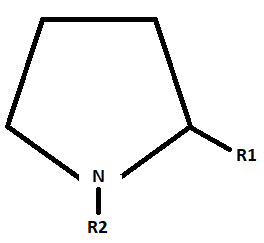** | **Pyrrolidinemethanol**: R1:CH_2_OH, R2:H  **1-(1-Cyclohexen-1-yl) pyrrolidine:**  R1:H, R2: Cyclohexene | 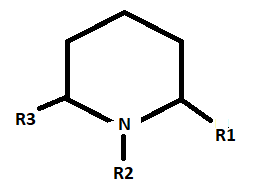 | **Methyl-6-propylpiperidine**: R1:CH3,R2:H,  R3:CH2CH2CH3  **1-Piperidineethanol**: R1, R3:H; R2:CH_2_OH | **3-Amino-4-pyrazolecarbonitrile**  **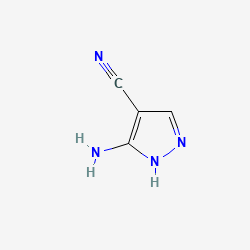** | 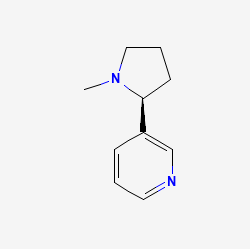**3-(1-Methyl-2-pyrrolidinyl) pyridine** | 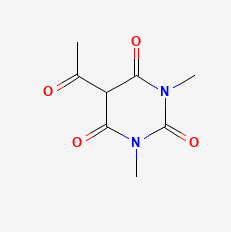**5-Acetylpyrimidine-2,4,6(1H,3H,5H)-trione** |
| --- | --- | --- | --- | --- | --- | --- |

**Alkaloids**

| **Decahydroquinoline**  **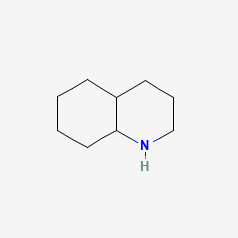** | 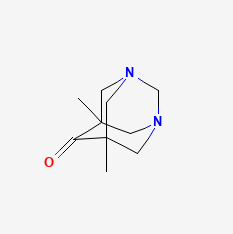**5,7-Dimethyl-1,3-diazadamantan-6-one** | 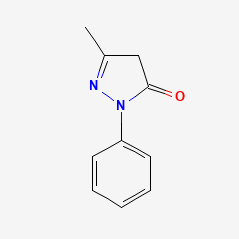**2,4-Dihydro-5-methyl-2-phenyl-3H-Pyrazol-3-one** | **44-Formyl-1,3-dimethyl-1,3(2H)-dihydroimidazole-2-thione**  **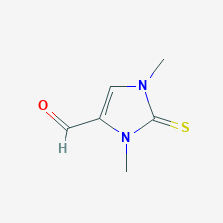** | 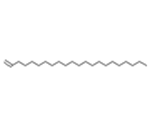**1-Docosene** |
| --- | --- | --- | --- | --- |

**Quinones**

| **Alpha-Lapachone**  **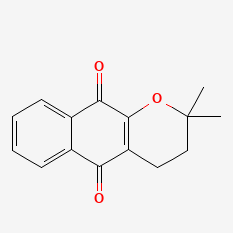** | 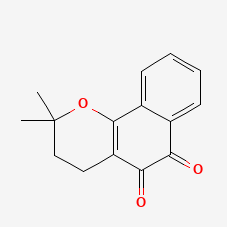**Beta-Lapachone** | 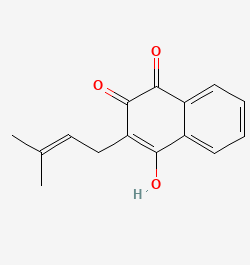**Lapachol** | 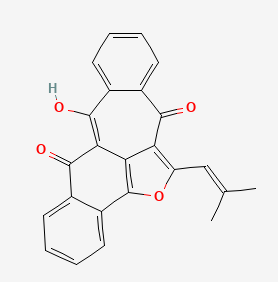**Radermachol** | 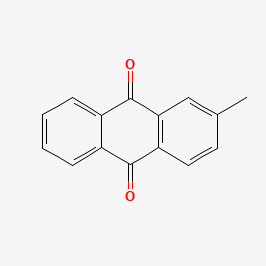**Tectoquinone** | 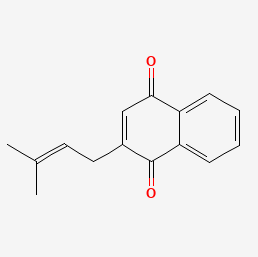**Deoxylapachol** |
| --- | --- | --- | --- | --- | --- |
| 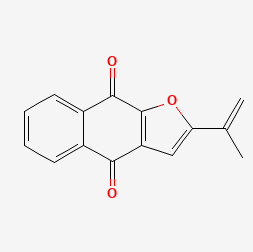**2-Isopropenylnaphtho[2,3-b]furan-4,9-quinone** | | 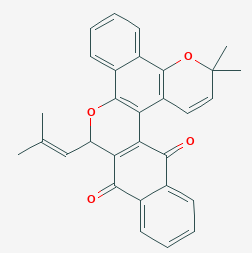**Tecomaquinone** | 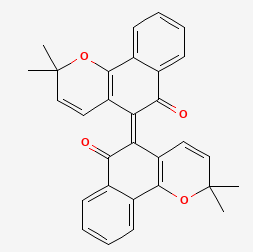**Dehydrotectol** | | |

**Terpenoids**

| 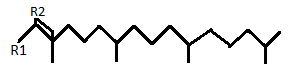  **3,7,11,15-Tetramethyl-2-hexadecen-1-ol**: R1:CH2OH,R2:H  **2-Hexadecene, 3,7,11,15-tetramethyl-, [R-[R*,R**-(*E)]]**-: R1:CH3,R2:H | **Betulinic Acid** | **Urs-12-ene** | **2,6,10-Trimethyl,14-ethylene-**  **14-pentadecane** | **Kauran-18-al, 17-(acetyloxy)-, (4.beta.)-** |
| --- | --- | --- | --- | --- |
|  | 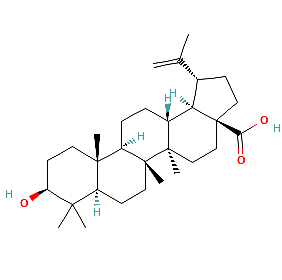 | 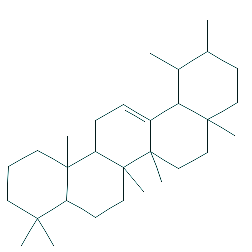 | 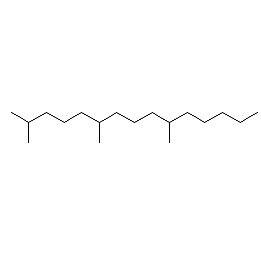 | 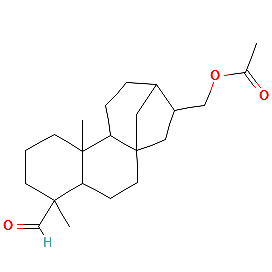 |
|  | **Olean-12-ene**  ***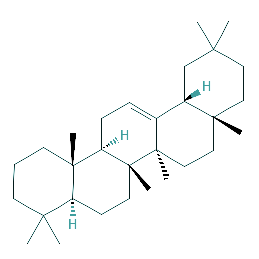*** | 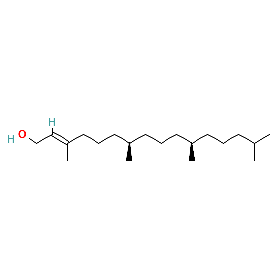**Phytol** | 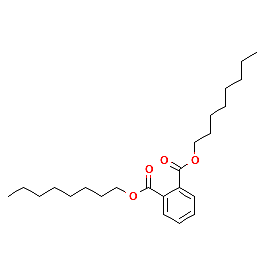**Di-n-octyl phthalate** – | 3**,7,11,15-Tetramethyl-2-hexadecen-1-ol**  **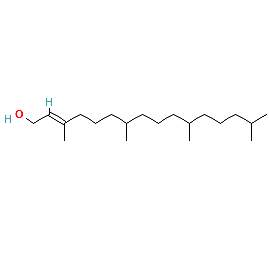** |

| **Fatty Acid, -esters, - aldehydes and -alcohols** | | | | | |
| --- | --- | --- | --- | --- | --- |
| **Myristyl aldehyde**  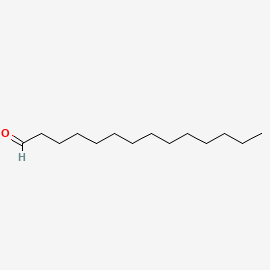 | 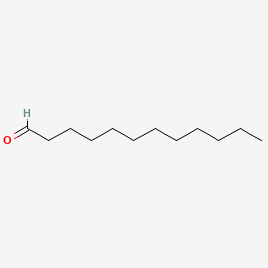**Dodecanal** | 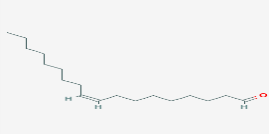**9-Octdecenal, (Z)** | **Octadecanal**  **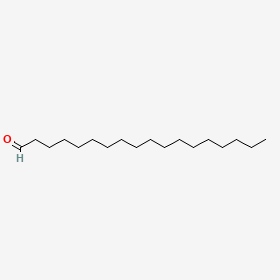** | **Cis-9-Hexadecenal**  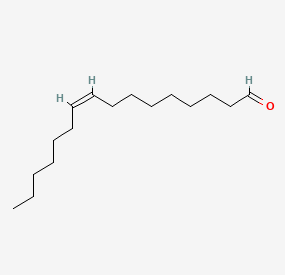 | **Palmitic acid**  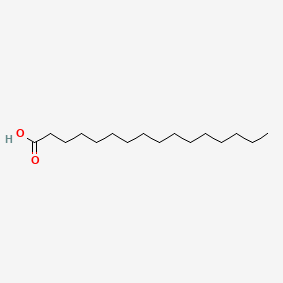 |
| **n-Hexadecanoic acid**  **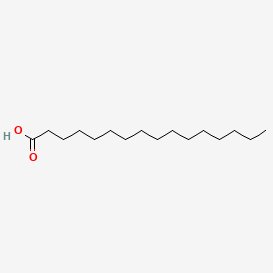** | **Hexadecanoic acid. ethyl ester**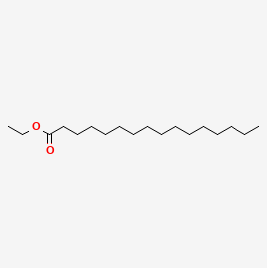 | 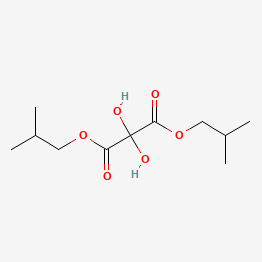**Di isobutyl 2,2-dihydroxy malonate** | **Octadecanoic acid, butyl ester**  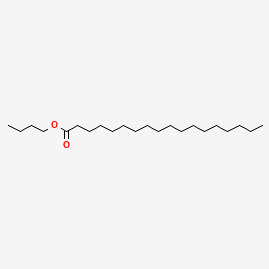 | **Hexadecanoic acid, butyl ester**  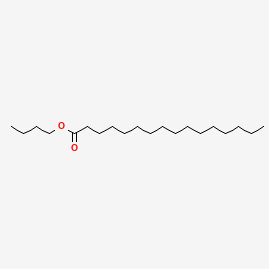 | **Stearic acid hydrazide**  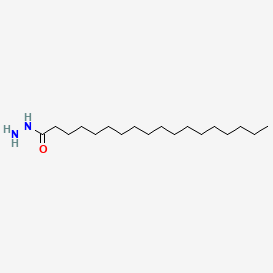 |
| **Ethyl-9-hexadecenoate**  **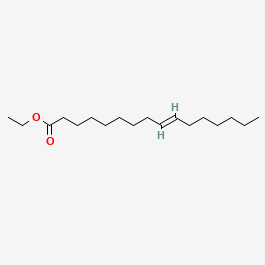** | 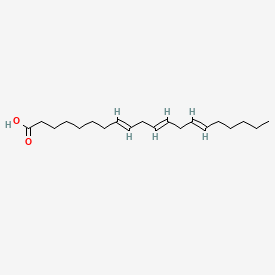**8,11,14-Eicosatrienoic acid, (Z,Z,Z)-** | **Eicosanoic ester, ethyl ester**  **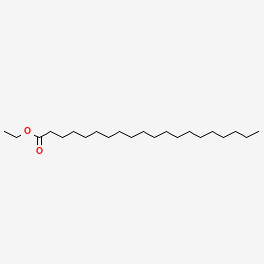** | **1-Heptacosanol**  **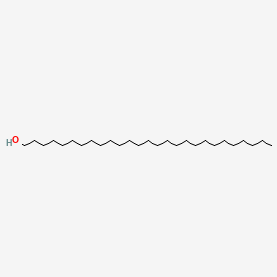** | **1-Undecanol**  **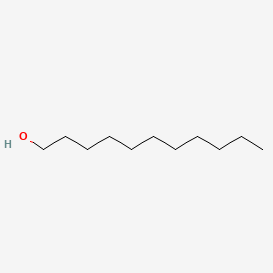** | **trans-2-Dodecen-1-ol,trifluoroacetate**  **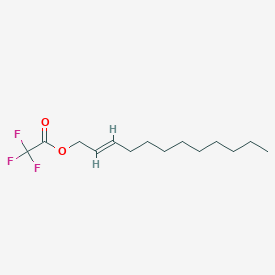** |

**Others**

| **Undulatin**  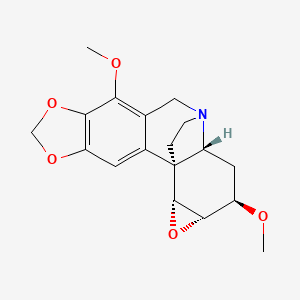 | **Cyclohexanepropanol**-  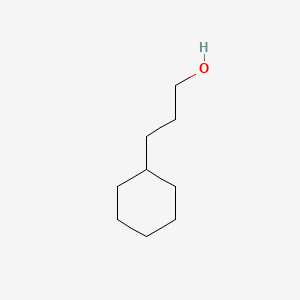 | **1,3-Isobenzofurandione, hexahydro**  **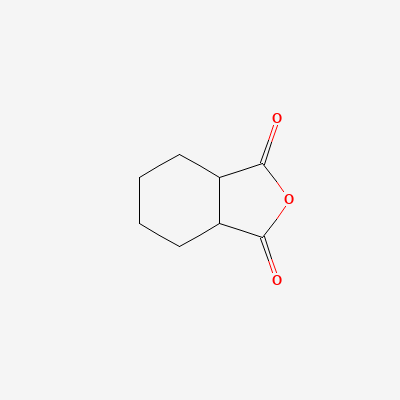** | **Heptadecyl heptafluorobutyrate**  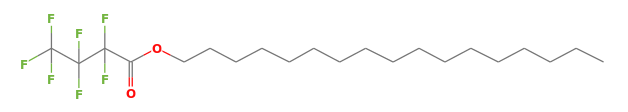 | 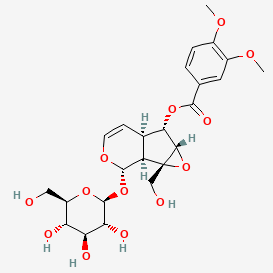**6-0-veratryl catalposide** |
| --- | --- | --- | --- | --- |
| 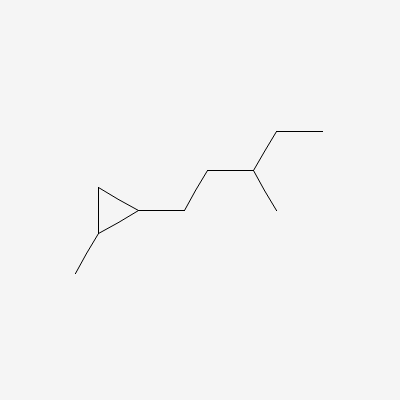**Cyclopropane, 1-methyl-2-(3-methylpentyl)-** | **3-Undecene, 7-methyl-, (E)-**  **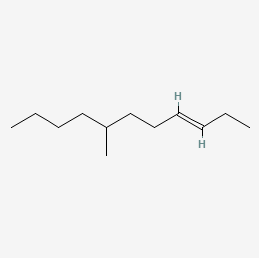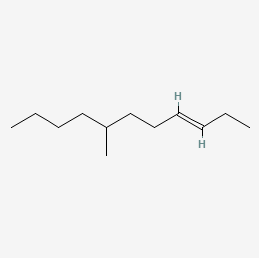** | **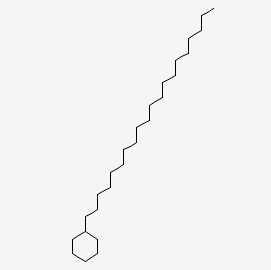Cyclohexane, eicosyl-** | **2-Nonadecanone**  **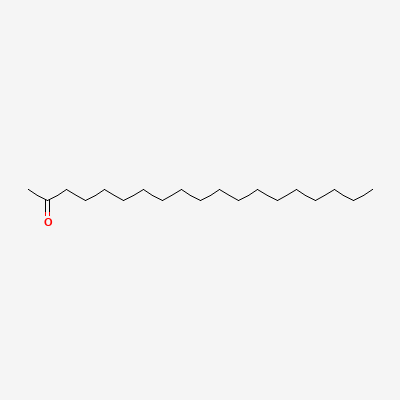** | **2-Undecene, 6-methyl-, (E)-**  **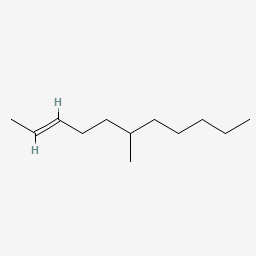** |
